# A Comprehensive Review on the Anticancer Activity of Plant Peptides and Their Mechanisms of Action

**DOI:** 10.3390/foods15091532

**Published:** 2026-04-28

**Authors:** Tianyu Hou, Yuanying Wang, Yulong Yao, Yangfan Hu, Vasudeva Reddy Netala, Huizhen Li

**Affiliations:** 1 School of Chemistry and Chemical Engineering, North University of China, Taiyuan 030051, China; yy18234732235@163.com (Y.W.); 15548807250@163.com (Y.Y.); 13934877042@163.com (Y.H.); vasunuc1922@gmail.com (V.R.N.); hzli@nuc.edu.cn (H.L.); 2 Zhendong Traditional Chinese Medicine, Zhendong Science and Technology Park, Changzhi 047199, China

**Keywords:** plant peptides, anticancer, apoptosis, angiogenesis, metastasis, cyclotides, defensins

## Abstract

Plant-derived peptides have become one of the most promising classes of compounds in cancer research due to their specificity, safety, and different therapeutic actions. Generally, plant peptides have a size of 2 to 100 amino acids, and they can be extracted from different parts of the plant including leaves, seeds, stems, and roots. The present review brings together more than 300 prominent plant peptides, their sources, structural classes, extraction methods, anticancer effects, and mechanisms of action. We show the cytotoxicity of plant peptides against a wide range of human cancer cell lines (such as MCF-7, A549, HL-60, and HCT-116), as well as their effectiveness in preclinical animal models of cancer, where they resulted in lesser tumor growth and metastasis. Moreover, we go into the anticancer activity of plant peptides and reveal the interconnectedness of apoptosis, cell cycle arrest, angiogenesis inhibition, metastasis suppression, and the modulation of signaling pathways as some of the mechanisms through which plant peptides perform. In addition to their therapeutic potential, many of these peptides are derived from edible plant sources and can be delivered through functional foods or dietary supplements, offering a promising avenue for cancer prevention and adjunctive nutritional support. The review also touches upon the major hurdles in peptide drug development at present, such as stability, oral bioavailability, and large-scale production, while at the same time giving future perspectives that include bioengineering, nanotechnology-based delivery systems, and combination therapies for translating these natural products into clinical oncotherapeutics and health-promoting foods

## 1. Introduction

**Figure 1:**
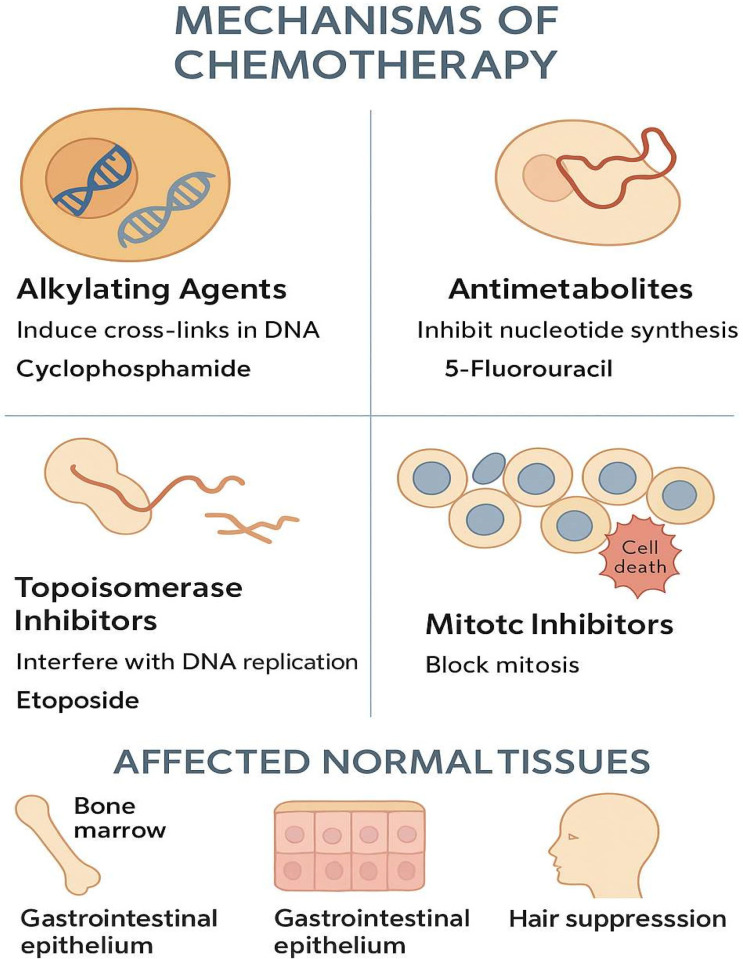
The four principal groups of chemotherapeutic drugs: alkylating agents (DNA cross-linking), antimetabolites (nucleotide synthesis blocking), topoisomerase inhibitors (DNA replication interfering) and mitotic inhibitors (cell division blocking). This group of drugs has the main effect on the rapidly dividing cells of cancer, which eventually leads to their death. However, everyday cells such as those in the bone marrow, GI tract, and hair follicles are also targeted and thus suffer from the same effects, i.e., side effects like nausea, anemia, and hair loss. (Figure is created using BioRender).

Cancer is not only a deadly disease but also a major health problem worldwide. In 2022, WHO estimated cancer cases to be around 20 million and deaths to be 9.7 million [1,2,3]. Cancer is a disease that involves not only human health but also worldwide economies, healthcare systems, and families [4,5,6]. It is a disease that is characterized by complexity, change over time, and resistance to treatment. Surgery, radiation, and chemotherapy usually help, although they already have their limits. These limits can both reduce the effectiveness of the treatment and affect the patient’s quality of life [7,8,9,10]. Chemotherapy agents that are currently in use destroy cancer cells but they also attack fast-dividing healthy cells in the process. Such healthy cells are located in bone marrow, the digestive tract, and hair follicles (see Figure 1). Consequently, patients suffer from serious adverse effects that include low blood counts, sores in the mouth and stomach, nerve pain, loss of hair, and extreme lethargy. All of these side effects lead to a reduction in the drug dosage or to the delaying of the treatment, which lowers the success rate of the therapy [11,12,13,14]. Moreover, cancer cells can develop resistance to multiple drugs, which is referred to as multi-drug resistance (MDR). In this case, cancer cells manage to escape the action of different chemotherapeutic agents. They might export the drugs out of the cell, modify the target of the drug, repair the damaged DNA, or survive the process of cell death. Thus, even strong chemotherapy combinations may end up being ineffective against these resistant cancer cells [15,16,17,18].

The plant kingdom is a great source of active compounds that have been used as the basis for modern day drugs. It is estimated that 40% of the drugs used in clinics today are either directly or indirectly from natural sources [19,20,21,22,23]. Notable examples include paclitaxel (from *Taxus brevifolia*), which stabilizes microtubules [24,25,26]; vincristine and vinblastine (from *Catharanthus roseus*), which inhibit microtubules; and camptothecin (from *Camptotheca acuminata*), a topoisomerase I-targeting agent.

The plant innate immune system has evolved to combat different types of attackers including pathogens, insects, and environmental stresses, and as part of it, produces a huge variety of peptide-based defense molecules. Some of the biological activities of the plant peptides include not only cytotoxicity but also antimicrobial, antifungal, antiviral, insecticidal, and immunomodulatory properties [27,28,29,30]. The plant peptides’ actions have many mechanisms which include the disruption of membranes, the inhibition of enzymes, the locking of receptors, and the promotion of cell death [28,29,30,31,32].

**Figure 2:**
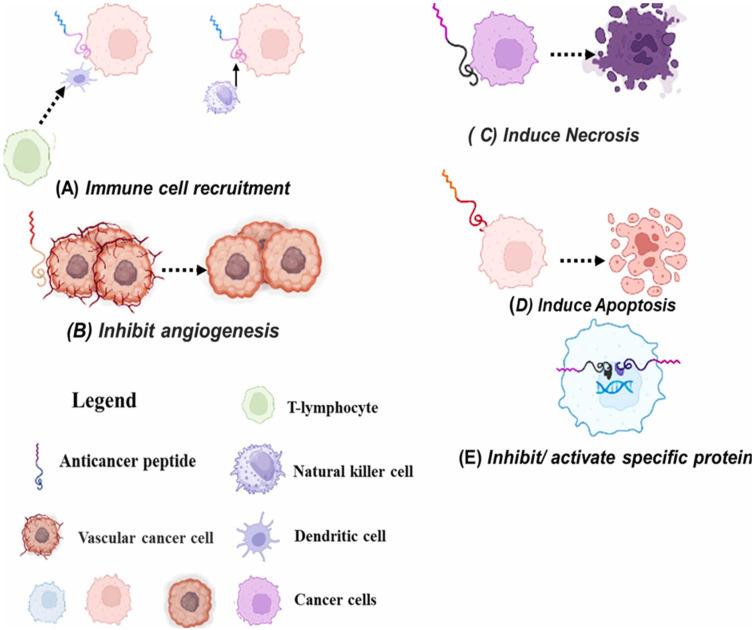
Multimodal anticancer mechanisms of PACPs. PACPs target tumors through five primary mechanisms: (**A**) Immune cell recruitment: (**B**) Inhibition of angiogenesis: (**C**) Induction of necrosis: (**D**) Induction of apoptosis: (**E**) Inhibition/activation of specific proteins. Figure is adapted from Chinnadurai et al. (2023), Open access article, Biomedicine and Pharmacotherapy Journal [39].

Plant anticancer peptides (PACPs) are usually small, positively charged and hydrophilic or hydrophobic molecules, and their cyclic forms are stabilized by the presence of a disulfide bond which in turn not only enhances their activity but also makes them resistant to the action of proteolytic enzymes. The positive charge of PACPs enables them to interact more vigorously with the negatively charged phospholipid head groups and glycoproteins (such as phosphatidylserine, O-glycosylated mucins, and heparin sulfates) that are present on the surface of cancer cells in large amounts and are therefore selectively exposed [33,34,35]. On the one hand, this electrostatic attraction occurs to a much smaller extent in the case of normal healthy cells with their neutral zwitterionic membranes, which are rich in phosphatidylcholine and sphingomyelin [36,37,38]. PACPs strike the tumors (Figure 2) in a number of ways, one of which is by immune cell recruitment (Figure 2A), whereby the PACPs act as chemoattractants, thus activating and recruiting the cells of the body’s immune system to the tumor site. The immune cells that have been attracted can then discern, assault, and aid in the elimination of cancer cells, thereby, in essence, enhancing the innate and adaptive immune response of the host against the tumor [39,40]. PACPs inhibit angiogenesis (Figure 2B). PACPs are directly targeting and thereby disrupt the process of forming new blood vessels (angiogenesis) that help the tumor grow. The peptide ensures that the tumor does not receive any oxygen and nutrients by cutting off the supply through the blood vessels, thereby greatly reducing its growth and spread [41]. PACPs cause necrosis (Figure 2C). PACPs directly attack the cancer cell membranes. PACPs cause the death of the cell part through apoptosis (Figure 2D). PACPs have the power to set into action the intricate internal suicide mechanism of a cancer cell [42,43]. Specific proteins are further inhibited/activated by the action of PACPs (Figure 2E): PACPs act by inhibiting the critical proteins responsible for cancer cell proliferation and survival and activate the proteins that kill tumors [44].

## 2. Plant Peptides: Sources, Extraction, Purification and Identification

Peptides from plants have been obtained from an extensive and diverse range of botanical sources including legumes such as soybean, chickpeas, white and mung beans, black soybean, and peas, along with other protein-rich pulses like lentils, fava beans, and lupini [44,45]. Cereals and grains also play a big part in the contribution, with examples such as corn, rice bran, barley, buckwheat, oats, and millet, as well as wheat, rye, and quinoa [46]. Nuts have been listed as a great source as well, among which the first ones are peanuts, walnuts, almonds, macadamia, cashews, pistachios hazelnuts, Brazil nuts, and so on [47]. Green leafy veggies including dendrobium, amaranthus, duckweed, and spinach, along with cressa cretica, kale, Swiss chard, and parsley, are among the top sources [48]. Oilseeds stand tall as the richest in peptides, with rapeseed, olive seed, amaranth, black seeds, chia, sunflower, flax, hemp, and pumpkin [49]. Marine plants are the most varied source, as the species include microalgae (spirulina and cyanobacterium) and macroalgae (Laver, kelp, sea lettuce), along with mangroves [50]. Finally, chamomile flowers, rice husk, tomato fruits, potato tubers, and seeds of pumpkin and brucea javanica are included [51].

**Figure 3:**
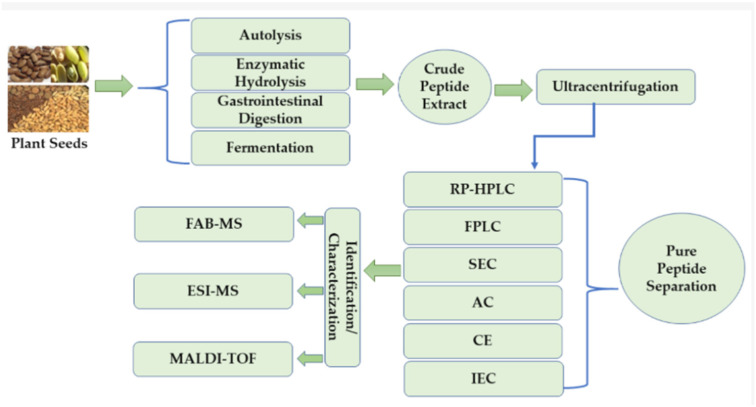
Pipeline for the extraction, purification, and characterization of PACPs. The schematic illustrates the sequential process from raw plant material through bioprocessing, separation, and final identification using chromatographic and mass spectrometric techniques. Figure is adapted from Samtiya et al. Open access article, Foods, MDPI 2021 [53].

The PACPs are isolated and characterized using various methods such as autolysis, enzymatic hydrolysis, simulated gastrointestinal digestion, and fermentation, which result in the breakdown of complex proteins into crude peptide extract (Figure 3). The ultracentrifugation is used for the purification of this complicated mixture by removing the insoluble materials [52,53]. The following purification step consists of an array of various chromatography techniques to achieve the separation of the peptides into pure fractions; these include RP-HPLC for hydrophobicity-based separation, FPLC, SEC for molecular size-based separation, Affinity Chromatography for specific binding interactions, Capillary Electrophoresis for charge and size-based separation, and IEC for net surface charge-based separation. The purified peptides are finally identified and characterized using advanced mass spectrometry methods, such as FAB-MS, ESI-MS, and MALDI-TOF, which help to determine their exact molecular weights and amino acid sequences, thereby confirming their identity and structure [54,55].

## 3. Different Extraction Methods of PACPs

To boost the enzymatic hydrolysis of parental proteins, different novel physical processing technologies are gaining more and more acceptance. The most important technological explorations related to this area include microwave-assisted extraction, ultrasound-assisted extraction, Pulsed Electric Field, hydrostatic high-pressure processing, subcritical water, and ohmic heating [56].

### 3.1. Microwave-Assisted Extraction (MAE)

MAE has proven to be a very effective approach, especially when used along with enzymatic hydrolysis. This method of extraction applies microwave radiation (300 MHz–300 GHz) to the natural materials so that it can release the bioactive compounds in them very quickly. Microwave radiation breaks hydrogen bonds, allows ions to move more freely in the liquid, and increases the permeability of the biological material [57]. The changed structure of the material unfolds the proteins and brings their cutting places to light, thus making them ready for the next step of being attacked by the protease. As a result of the microwave pre-treatment, there are remarkable benefits when compared to the traditional methods. It not only shortens the processing time drastically but also increases the hydrolysis efficiency, which in turn leads to a higher proportion of low MW peptides and the final protein hydrolysate being more bioactive. Moreover, its environmentally friendly aspect, together with its operational simplicity, easy handling, and low cost, make MAE a mostly favored option for the development of plant peptides with bioactivity [58,59].

### 3.2. Ultrasound-Assisted Extraction (UAE)

**Figure 4:**
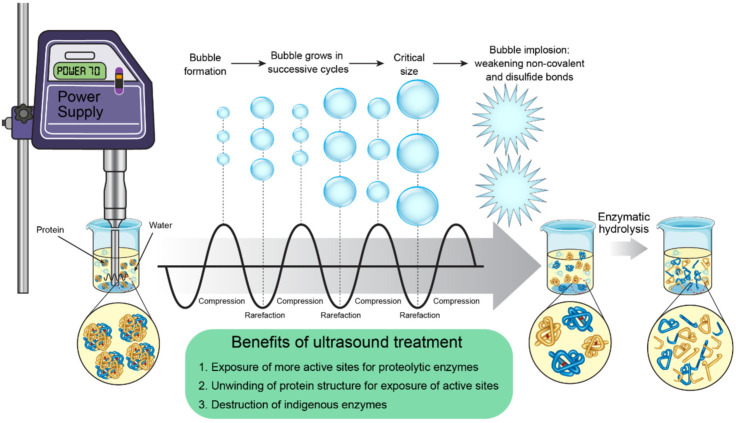
Mechanism of ultrasound-assisted protein hydrolysis. The figure illustrates the principle of cavitation induced by ultrasonic sound waves. A power supply generates waves that create cycles of compression and rarefaction in a solution, leading to the formation, growth, and eventual implosion of bubbles. The extreme physical forces from bubble implosion weaken non-covalent and disulfide bonds in proteins. This process unfolds the protein structure, exposing active sites for enhanced enzymatic attack, accelerates the hydrolysis reaction, and can deactivate undesirable indigenous enzymes. Figure is adapted from Fadimu et al. Open access article, Foods, MDPI, 2022 [60].

UAE is an eco-friendly and non-thermal physical technology used extensively for improving the extraction of PACPs from plant proteins (Figure 4). The UAE method utilizes high-frequency sound waves, normally in the range of 20 kHz, which are produced by a power supply and transmitted through a liquid medium. The sound waves are basically alternating compression and rarefaction cycles that, in turn, lead to the birth, expansion, and finally the popping of microscopic bubbles, which is called cavitation. The very intense collapse of these bubbles brings about the release of equally strong shear forces which act upon proteins and in doing so pull apart the non-covalent interactions and disulfide bonds that are established in the protein structure [60,61]. The strength of UAE lies in the fact that it can ‘induce’ proteins to go through drastic conformational changes, like ‘melting’ and an increase in β-sheets and β-turns. This change in molecular structure degrades the protein matrix, thus allowing the native structure to unwind and subsequently the active and cleavage sites that were originally hidden from view to be exposed. In consequence, enzyme penetration to the peptide bonds is markedly increased, which leads to the hydrolysis rate being very much accelerated and the yield of plant peptides being very much increased. Moreover, the energy produced from cavitation can at the same time wipe out the native enzymes that might be responsible for the formation of undesired products; thus, product purity is increased [62,63,64]. Ultrasonic processing is one of the pretreatment methods that has many advantages, such as very fast heating up and mass transfer, a shorter processing time and lower temperature, excellent process control, better extraction selectivity, quick start-up, easy installation, and overall operational simplicity [60,61,62,63,64].

### 3.3. Pulsed Electric Field (PEF) Technology

Pulsed Electric Field (PEF) technology, a revolutionizing non-thermal way of getting peptides released from food sources, is the application of high-voltage short-duration electric field pulses of 10 to 80 kV/cm at the micro- or millisecond level (not to be confused with the continuous field method). The effect of the electric field on the proteins is that it causes the proteins to get their non-covalent and covalent bonds broken, resulting in the collapse of their 3D structure (unfolding, denaturation, or gelation, depending on the conditions used). This in turn makes proteolytic enzymes more able to attack the proteins at their sites, thereby increasing the yield of peptide extraction [65,66]. Among the aspects in favor of PEF are its short treatment time, low energy consumption, ability to kill microorganisms, and the retention of heat-sensitive compounds, which is the main advantage of non-thermal treatment. However, the technology still has to deal with issues such as very high capital investments for the first time and safety concerns over the electrochemical reactions occurring at the commonly used electrodes (e.g., stainless steel), which can lead to metal ionization and product contamination. These limitations are what currently prevent widespread industrial use, and even in that case, research directed toward the perfecting of electrode materials and system design will continue [65,66,67].

### 3.4. High Hydrostatic Pressure (HHP)-Assisted Extraction

High Hydrostatic Pressure (HHP)-Assisted Extraction is a new, non-thermal technology which has been developed as a pretreatment method to increase the production of plant peptides. This batch process uses a high-pressure pump to push water—the pressure-transmitting fluid—into a top-notch high-pressure vessel which is then controlled by heating/cooling systems for the desired temperature. The protein sample is in this medium, and pressures between 100 and 1000 MPa are applied uniformly in all directions, with or without additional heat. After the process, the valve opens to release the pressure very quickly, and the sample is taken out [60,68]. The huge pressure causes very deep changes in the proteins’ structures: it breaks non-covalent interactions (e.g., hydrogen bonds, hydrophobic forces) and covalent disulfide bonds, leading to the unfolding of tertiary structures and disruption of secondary elements (e.g., alpha-helices, beta-sheets). Such deformation exposes buried active sites and cleavage points, thus greatly enhancing protein digestibility. HHP pretreatment along with enzymatic hydrolysis speeds up the rupture, producing more short-chain plant peptides that not only have higher purity but bioactivity as well [68,69]. Seen as a clean technology, HHP possesses benefits such as low power consumption, rapid pressurization/depressurization, a short processing time, and little effect on heat-sensitive compounds [60,68,69,70].

### 3.5. Ohmic Heating Assisted Extraction

**Figure 5:**
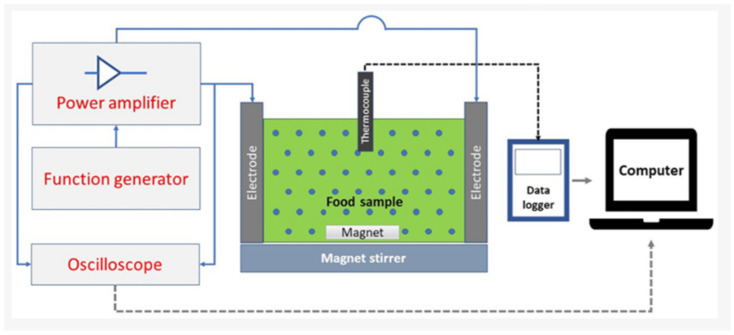
Schematic representation of an ohmic heating system for bio-peptide extraction. The diagram illustrates the key components of the setup: a function generator and power amplifier that produce and amplify an alternating current, which is applied via electrodes to the food sample. A thermocouple monitors temperature in real time, while a magnetic stirrer ensures uniform heating and mixing. An oscilloscope and data logger connected to a computer enable precise control and monitoring of electrical parameters and temperature profiles. Ohmic heating uses electrical resistance to generate rapid, uniform heat, enhancing protein denaturation, improving membrane permeability, and facilitating the release of plant peptides while minimizing thermal degradation. Figure is adapted from Safarzadeh Markhali et al., 2022, Open access article, Clean Technologies, MDPI [71].

Ohmic heating is an innovative thermal processing technology that could be an alternative method for the production of plant peptides (Figure 5). It can be considered a novel method as it uses the very nature of the food sample, that is its electrical resistance, to heat up the food very fast and uniformly. The system’s main components include a function generator that generates an alternating current, a power amplifier that boosts the signal, electrodes that feed the current into the sample, and a data logger linked to a computer for monitoring and control in real time. A thermocouple monitors temperature, while a magnet and a magnetic stirrer keep the mixing and heat distribution uniform [71]. The electric field tension raises the electric conductivity and makes the cell membranes more porous, thus astoundingly increasing the extraction rates of biomolecules, especially in thick or particle-rich fluids. The rapid and even heating with little thermal degradation achieved by Ohmic heating retains the nutrients, flavors, and quality. On top of that, it encourages protein unfolding, denaturation, aggregation, and gelation, and thus the release of free amino acids happens soon; moreover, the enzyme used in the second stage for peptide production works more efficiently due to the aid of hydrolysis [71,72,73]

While all the above-mentioned physical processing technologies enhance peptide yield and bioactivity, their readiness for industrial food applications varies considerably. Ultrasound-assisted extraction (UAE) and microwave-assisted extraction (MAE) are currently the most promising for the food industry due to their relatively low capital cost, operational simplicity, scalability, and established use in existing food processing lines [57,58,59,60,61,62,63,64]. Both are compatible with batch and continuous modes, have short processing times, and are considered environmentally friendly. Ohmic heating is also gaining industrial traction, particularly for liquid and semi-solid food matrices, because it enables rapid, uniform heating with minimal thermal degradation, though electrode material and fouling remain challenges [71,72,73]. High hydrostatic pressure (HHP) is already commercialized for pasteurization and sterilization, and its adaptation for peptide extraction is feasible; however, its high capital investment and batch-only operation limit its widespread adoption for routine peptide production [68,69,70]. Pulsed electric field (PEF) and subcritical water extraction are highly effective at laboratory scale and show great potential, but they are still constrained by high equipment costs, electrode corrosion (PEF), and the need for specialized pressure vessels (subcritical water). Therefore, for the functional food industry, UAE and MAE represent the most immediately deployable and cost-effective technologies, whereas PEF and HHP are better suited for high-value nutraceutical or pharmaceutical peptide production where higher investment can be justified [56,65,66,67].

## 4. Structural Features of PACPs

The structural diversity of PACPs has a major impact on their function and stability. These peptides are characterized mainly according to their most important secondary and tertiary structures, which, in turn, determine their interaction with and ability to kill cancer cells. The four main structural classes are listed below.

### 4.1. α-Helical Structure

**Figure 6:**
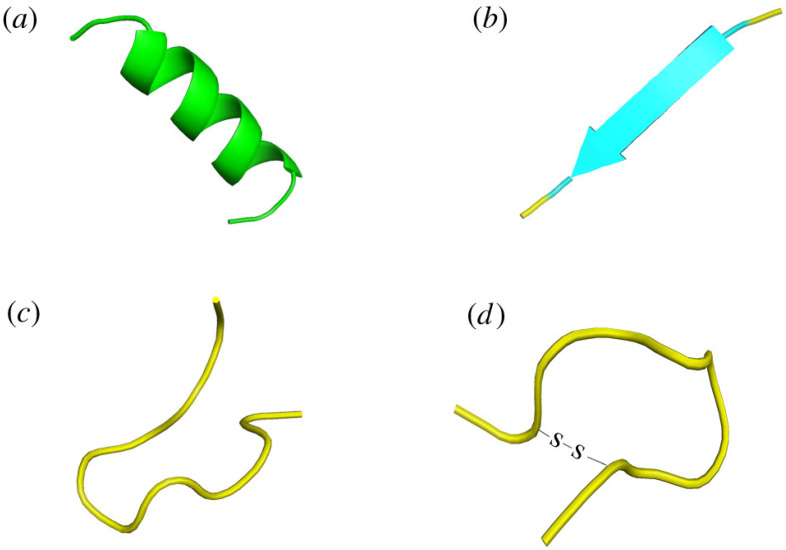
Structures of PACPs (**a**) α-helical; (**b**) β-pleated sheets; (**c**) random coil; (**d**) cyclic ACPs. Figure is adapted from Xie et al. 2020, Open access article, Open Biology Journal [74].

A major category has been characterized by its rod-like, coiled structure called α-helix (Figure 6a), the role of which is played by hydrogen bonds aligned parallel to the helix’s axis that confer stability. Such peptides are usually very short, straight, and have a basic but very functional amphipathic design; this implies that one end of the helix is water-repellent while the other is water-attracting, which is an important characteristic for the primary mechanism of action, the so-called recognition and consequent disruption of the distinctive membranes of cancer cells. The simplicity of such structures facilitates their use as standard templates for both de novo synthesis and modification in laboratories. An excellent example of α-helical structure peptides is plant thionins [74,75].

### 4.2. β-Pleated Sheets

On the other hand, the second type has an intricate and strict architecture that the peptide chain winds into strands held together by hydrogen bonds, creating wide and flat formations like sheets (Figure 6b). One of the main features of these peptides is that they are made up of several disulfide bonds which act like strong links connecting the cysteine residues and thus forming a stable and often cyclized core skeleton. The complexity of this structure gives them a very high resistance to proteolytic degradation and also very high thermal stability, which in turn allows them to survive and function even in very extreme physiological conditions where their anticancer action can be through the disruption of membranes or intracellular targets. It is stated that they are in the plant defensins category of β-pleated sheet structures [76].

### 4.3. Random Coil Structure

The next group possesses an innate quality of being flexible to the point of not having a consistent and definite shape at all, rather living in a state of disorder and continual change (Figure 6c). The presence of amino acids high in proline, which hinders the formation of helices, and glycine, which grants utmost flexibility, often contributes to the randomness of their structure. Their biological activity does not rely on adopting a specific fixed conformation, which means they can easily adjust themselves and interact with a broad spectrum of molecular targets, often through mechanisms related to immunomodulation like stimulating natural killer cells instead of direct membrane lysis [77].

### 4.4. Cyclic Peptides

As a conclusion, the last class which is the most robust structurally consists of the peptides that build a closed wire, thus eliminating the weak terminal ends (Figure 6d). Cyclization can be achieved either via the formation of a direct covalent bond between the amino and carboxyl termini of the peptide (head-to-tail method) or by building a network of disulfide bonds that produce a knotted core as stable as the former. The drawing of this structure gives it an exceptionally high strength that enables it to resist enzymatic breakdown and proteolysis in the bloodstream, which in turn greatly increases their therapeutic applications. The limitation in their shape also permits very selective and strong interactions with the respective cellular targets; hence, they are the main focus of clinical studies. Most plant peptides belong to this class, with the best example being cyclotides [78].

## 5. Plant Anticancer Peptides and Their Mechanisms of Action

Lunasin, a peptide that occurs naturally, has advanced gradually in the field of epigenetics, and its chemopreventive action has become recognized as very powerful. The first source identified was soy, but research on it yielded more discoveries and finally incorporated it among several plant seeds edible for humans, thus marking its role in functional foods. The pioneering study of Galvez et al. [79] was one of the first to give a detailed description of the peptide lunasin, which is a 43-amino-acid peptide with a unique molecular structure: a polyaspartic acid tail, an Arg-Gly-Asp (RGD) cell adhesion motif, and a presumed chromatin-binding helix. The study also demonstrated for the first time that lunasin peptide used exogenously could inhibit the transformation of murine fibroblast cells induced by chemical carcinogen. In addition, crucially, it proposed the epigenetic mechanism which is still central to the function of lunasin: the peptide enters the cells through the RGD motif, binds to deacetylated histones with high affinity and prevents the acetylation of core histones H3 and H4. This mechanism is also responsible for its selective toxicity, since lunasin caused apoptosis in oncogene-transformed cells but did not affect non-transformed ones. The in vivo effectiveness of lunasin was established using a model of skin cancer in SENCAR mice where topical application resulted in a decrease of about 70% in tumor incidence [79].

The research conducted by Luna-Vital et al. [80] represents a major advance in our knowledge of the anticancer effect of common bean (*Phaseolus vulgaris* L.) peptides, corresponding to colorectal cancer chemoprevention. As a result of the study, five main peptides were recognized, namely GLTSK, LSGNK, GEGSGA, MPACGSS, and MTEEY, which the researchers believe to be responsible for considerable anticancer effects against colorectal cancer cells. Moreover, the cultivar affected both the potency of the peptides and the mechanism of action. Peptides from the ‘Azufrado Higuera’ cultivar displayed the highest potency against HCT116 cells and were, therefore, proposed to be able to modulate the p21 and cyclin B1 cell cycle regulators. On the other hand, peptides from the ‘Bayo Madero’ cultivar demonstrated maximum efficiency against RKO cells and primarily interacted with the intrinsic apoptotic pathway by controlling the expression of the BAD, cytochrome c, and caspase-3 proteins. This suggests that the use of bean-derived peptides could result in the inhibition of tumor cell growth through two distinct but interlinked approaches: the stopping of cell cycles and the triggering of apoptosis through mitochondria [80].

**Figure 7:**
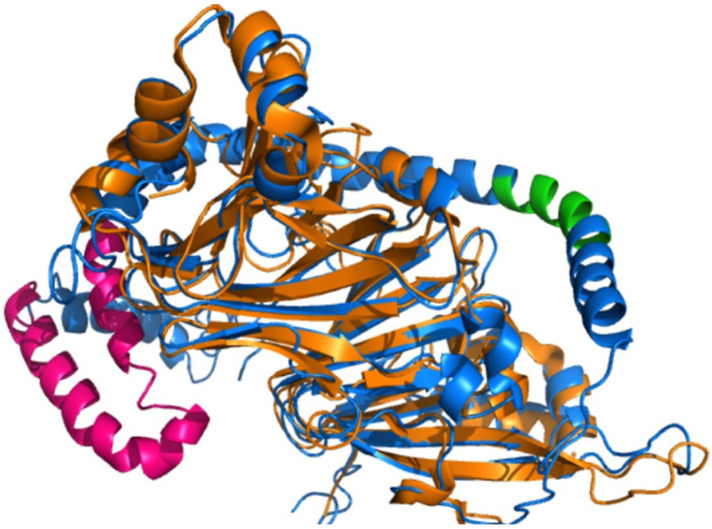
Three-dimensional structure of β-conglycinin regions containing ACP candidate sequences. Figure is adapted from Freitas et al. 2019, Open access article, Journal of Functional Foods [81].

The study conducted by Freitas and coworkers aimed to recover and characterize encrypted peptides with antimicrobial and anticancer activity from a protein-rich soybean meal (Glycine max) by-product. They prepared the aqueous extract with environmentally friendly methods, then used gel filtration chromatography for fractionation and finally identified two phenolic-reduced fractions (F1 and F2) as the most bioactive. Mass spectrometry analysis revealed that the fractions were a mixture of peptides that were mainly encrypted in the β-conglycinin alpha and alpha-prime subunits, and their molecular masses ranged from 718.42 to 4872.43 Da (Figure 7). By utilizing predictive algorithms, the researchers successfully recognized twelve peptides as the top antimicrobial candidates. The sequences of the key peptides were LSSTSEK, PRPIPFPRPQP, PRPIPFPRPQPQ, PRPIPFPRPQPQSQ, PRPIPFPRPQPQSQP, PRPIPFPRPQPQSQPS, PRPIPFPRPQPQSQPSQ, EPEAEEPEAEE, EPEAEEPEAEEI, EPEAEEPEAEEIE, and EPEAEEPEAEEIEA. These peptide mixtures, on the other hand, were found to be highly effective against human glioblastoma (U-87 MG) cells, and the selectivity was such that they managed to arrest the proliferation of cancer cells (IC_50_ 6.74 µg/mL for F1 and 2.57 µg/mL for F2) while having no lethal effect on healthy mouse bone marrow and fibroblast cells. Molecular modeling studies have shown that the bioactivity of peptides is located in the native β-conglycinin structure, which is in an alpha-helix conformation. This research reveals the potential of using soybean meal by-products as a sustainable source of multifunctional peptides for natural food preservatives and chemopreventive ingredients [81].

Soares et al. (2015) mentioned four peptides, including GGV, IVG, LVG, VGVI, and VGVL, obtained from *Amaranthus cruentus*, and initially investigated the hypocholesterolemic effect through the HMG-CoA reductase inhibition, but a very interesting indirect link to the cancer treatment area comes out of this mechanism as the mevalonate pathway, where these peptides act, is also the metabolic route for isoprenoids such as geranylgeranyl pyrophosphate production, which is one of the key metabolites for many tumors; on the other hand, the statins inhibiting HMG-CoA reductase have been clinically associated with cancer prevention via various epidemiological and preclinical models, and the peptides GGV, IVG, and VGVL—due to sharing the same inhibition of enzyme action—could potentially be investigated for the same anticancer effect in future research, although this is still a speculative yet scientifically possible extension of their reported bioactivity [82].

Hemp peptides (HP), derived from hemp seeds, have been shown by Wei et al. [83] to exert their strong anticancer effect on Hep3B liver cancer cells by acting through a dual mechanism. Firstly, the HP-treated cells experience a marked increase in the amounts of intracellular and mitochondrial reactive oxygen species (ROS), resulting in oxidative damage and hence cell death. Secondly, HP blocks the pro-survival Akt/GSK3β/β-catenin signaling pathway. This is done by turning off Akt, which in turn allows GSK3β to carry out the phosphorylation of β-catenin and mark it for degradation. The deactivation of β-catenin leads to the suppression of oncogenic genes. The combined effect of these actions is that the mitochondrial apoptosis pathway is turned on, which is indicated by an increase in pro-apoptotic Bad, a decrease in anti-apoptotic Bcl-2, and the cleavage of caspase-3; all these processes lead to the death and lessening of the movement of the cells. What is most important, though, is that this toxicity is only for the cancer cells, as the normal liver cells are not affected [83].

Kannan et al. [84] managed to separate and characterize an anti-cancer peptide that was novel from a defatted rice bran enzymatic hydrolysate that did not have more than a 5 kDa molecular weight. By means of a multi-stage purification method that was instrumental in identifying a pure pentapeptide with a molecular mass of 685.378 Da and amino acid sequence of Glu-Gln-Arg-Pro-Arg, they applied ion-exchange chromatography and HPLC for the purification process. This new peptide was able to kill cells and was not restricted to one particular type; hence, it was referred to as broad spectrum. When the concentration of the novel peptide was 600–700 μg/mL, it resulted in significant growth inhibition: 84% in colon cancer cells (Caco-2, HCT-116), 80% in breast cancer cells (MCF-7, MDA-MB-231), and 84% in liver cancer cells (HepG-2). The primary mechanism of action of this pentapeptide obtained from rice bran has been reported to be the induction of cancer cell growth arrest, which effectively blocked the proliferation. The researchers therefore concluded that this peptide might be a great nutraceutical candidate for cancer prevention and treatment [84]. Xue et al. [85] on the other hand showed that the chickpea peptide CPe-III-S (RQSHFANAQP) has a remarkable ability to bring about the death of breast cancer cells. The peptide remarkably stopped the growth of two different cell lines: MCF-7 (estrogen receptor-positive) and MDA-MB-231 (triple-negative) with EC50 values of 2.38 μmol/mL and 1.50 μmol/mL, respectively, meaning the activity was highly potent. The major mechanism of action that was discovered was the upregulation of the p53 tumor suppressor protein in a dose-dependent manner. Molecular docking studies indicated that the CPe-III-S peptide interacts with the p53 protein through a specific binding, which implies that this binding may render p53 stable or, even better, activate it. The increase in the functional p53 level then results in cancer cell proliferation being inhibited [85].

Kuerban et al. [86] have reported the extraction of bioactive peptide fractions (<3 kDa) from red and brown lentil (Lens culinaris) proteins through tryptic hydrolysis followed by ultrafiltration. The chromatographic separation of the peptide fractions was then complemented by HPLC-MS/MS identification, leading to the discovery of 28 novel peptide sequences. Thereafter, the research indicated that the peptide fractions from lentils had marked anti-cancer activity against a selected human cancer cell line. Among those, the prostate cancer cell line (PC3) was the least resistant and showed the strongest anticancer response with a corresponding very low IC_50_ value of 0.96 mg/mL. Sidestepping lung (HepG2) and breast (MCF-7) tumors is among these peptides’ accomplishments in limiting cancer cell proliferation [86]. Karami et al. [87] proved that the hydrolysates of defatted wheat germ protein had a concentration-dependent effect and a significant reduction in the viability of A549 human lung cancer cells. They managed to identify, by means of the nano-LC/ESI-MS/MS analysis, different peptides responsible for this anticancer effect such as KELPPSDADW and SSDEEVREEKELDLSSNE from a pepsin hydrolysate, TVGGAPAGRIVME, VGGIDEVIAK, and GNPIPREPGQVPAY from an Alcalase hydrolysate, and SGGSYADELVSTAK and MDATALHYENQK from a proteinase K hydrolysate. Out of these, SSDEEVREEKELDLSSNE was pointed out as having the most powerful cytotoxic activity [87].

The research conducted by Li et al. [88] revealed that the use of a papain hydrolysate derived from mung bean protein resulted in marked anticancer activity through both in vitro and in vivo models. In mice, the hydrolysate was able to stop the spreading of cancerous cells without causing any of the adverse effects that come with chemotherapy and was also able to trigger apoptosis and halt the cell division cycle (in the S phase for low doses and G0/G1 phase for high doses) for human liver cancer HepG2 cells. Once the ultrafiltration and Sephadex G-15 gel chromatography purification steps were completed, the most active fraction (Fraction A, <3 kDa) was able to completely rule out 86.35% of the HepG2 cells at the concentration of 2.99 mg/mL. The UPLC-MS/MS analysis that followed on this fraction uncovered the existence of four new peptide sequences that are responsible for the activity: VEG, PQG, LAF, and EGA. These peptides turned out to be composed mainly of hydrophobic amino acids (Gly, Ala, Val, Ile) and also Glu, Asn, and Gln. The main anticancer mechanisms that were discovered were the induction of apoptosis and cell cycle arrest, which demonstrates the great potential of these peptides derived from mung beans, not only as functional food components in the treatment of liver carcinoma cancers, but also as a source of natural food ingredients [88].

The findings of Hwang et al. [89] indicate that Chungkookjang, which is a Korean fermented soybean product, consists of a large number of peptides. These peptides, together with other constituents, were the reason for the concentration-dependent inhibition of breast cancer MCF-7 cell growth. The anticancer effect of the fermented soybean extract, which was at least in part due to its peptide content, was associated with profound changes in gene expression. DNA microarray analysis showed that the treatment upregulated major tumor-suppressive genes in the TGF-β pathway, such as TGFβ1 and Smad3. At the same time, it downregulated pro-inflammatory cytokines and their receptors (e.g., CSF2, CSF2RA and CSF3) and modulated the expression of chemokines (CCL2, CCL3, CXCL1, CXCL2). Network analysis put ERβ in this regulatory network. Thus, Chungkookjang peptides are thought to exert their anticancer effect through a dual mechanism: activation of the TGF-β/Smad3 tumor-suppressive pathway coupled with the resolution of cancer-promoting inflammation, which together hinder the proliferation of breast cancer cells [89].

Zheng et al. [90] extracted a powerful peptide mixture known as Fraction A3 from Dendrobium catenatum Lindley. This fraction selectively targeted and destroyed human cancer cells without harming healthy ones. They obtained it through enzymatic breakdown and purification by chromatography. At a concentration of 500 μg/mL, it inhibited cancer cell growth by 86.8% in breast cancer cells (MCF-7), 78.91% in gastric cancer cells (SGC-7901), and 73.38% in liver cancer cells (HepG-2). In contrast, it caused only slight damage (5.52% inhibition) to normal human liver cells (L-O2). Mass spectrometry identified three main peptides in this fraction: RHPFDGPLLPPGD, RCGVNAFLPKSYLVHFGWKLLFHFD, and KPEEVGGAGDRWTC. Synthetic versions of these peptides also showed anticancer activity. The peptides worked by selectively killing cancer cells without harming normal cells, suggesting they could be developed into therapies or nutraceuticals [90].

Tanya et al. [91] demonstrated that protein hydrolysates specifically plant peptides released from amaranth seeds (*Amaranthus caudatus*) during simulated human digestion exhibit potent anticancer activity against aggressive triple-negative breast cancer cells. This great effect was observed when digestion followed the heat denaturation of the proteins first; this process opens their structure up and helps the digestive enzymes in releasing a much larger amount of these therapeutic peptides. Peptides from amaranth are two main ways to combat cancer: they cause apoptosis and block metastasis. Apoptosis was established by a variety of characteristic cellular events including DNA fragmentation and condensation, cell membrane integrity loss, phosphatidylserine externalization and caspase-3 activation. Additionally, tumor cell migration was considerably inhibited in the cancer cell line treated with the peptides in a wound-healing experiment, thus revealing the strong possibility of preventing cancer from spreading. One of the reasons for this anticancer effect is the high antioxidant activity of the peptides, which scavenges the free radicals that could cause cancer proliferation [91]. Quinoa protein peptides such as IFQEYI, DVYSPEAG, RELGEWGI, DKDYPK, and LWREGM showed inhibition against colon cancer cells, highlighting that the digested quinoa protein is a rich source of anticancer peptides; the researchers proposed its use in the production of functional foods or nutraceuticals targeting cancer prevention [92].

The peptide from olive seed LLPSY exhibits a marked anti-proliferative effect by very markedly inhibiting the growth of cancer models that are particularly aggressive, namely MDA-MB-468 triple-negative breast cancer and PC-3 androgen-independent prostate cancer cell lines, thus showing the potential use of the peptide against treatment-resistant cancers. The antitumor action of the compound is, by its very nature, a complex one: it is a principal factor of tumor cell migration inhibitory power, possibly by cytoskeletal destabilization or MMPs suppression, while at the same time it is characterized by a significant increase in cellular adhesion to the extracellular matrix, which might be through the activation of the expression of E-cadherin and similar proteins; all this working together can bring down the metastatic potential effectively. On top of this, LLPSY causes strong cell cycle arrest exclusively in the S-phase by acting on the DNA replication process, it might be through the regulation of cyclins, CDKs, or checkpoint proteins like Chk1/2, and this would result in the synthesis of DNA being inhibited and thus the loss of cell division [93].

Rapeseed peptide (RSP), made from the hydrolysis of rapeseed protein, has an excellent anticancer effect on human cervical carcinoma (HeLa) cells by blocking the growth and triggering the process of apoptosis. RSP activates the typical apoptotic phenotypic alterations, leads to DNA damage, and facilitates cell cycle stoppage during S phase. The findings indicate that the RSP primarily acts as an antitumor agent through the induction of apoptosis. This research, being the first of its kind, not only sets the stage for further studies but also clears the pathway for establishing a direct cause–effect link between RSP treatment and apoptotic cell death in a classic cancer model. The discovery of S-phase arrest is of great importance as it points to a particular mechanism wherein RSP may disrupt the DNA replication process, possibly by blocking ribonucleotide reductase or incurring replication stress, thereby triggering the observed DNA damage and subsequent apoptotic cascade. This two-fold action actively blocking cell cycle progression and concurrently leading to cell death renders RSP a very attractive multi-targeted therapeutic candidate [94].

The study by Xue et al. [95] greatly contributed to the understanding of rapeseed meal’s anticancer effects by identifying a peptide fraction named RSP2 with potent activity against HeLa human cervical cancer cells. The results showed that RSP2 treatment caused a remarkable decrease in HeLa cell viability and that this effect was dose-dependent. The RSP2-treated cells also exhibited features of dying cells like being less round and changing their shapes. After conducting mechanistic studies, the researchers were able to conclude that RSP2 actually causes cells to die; this was supported by the occurrence of DNA fragmentation and the results of the comet assay, which indicated dose-dependent DNA damage. In addition, the inference drawn from the results of the cell cycle analysis was that RSP2 treatment significantly reduced (from 55% to 43%) G0/G1 phase cells and dramatically increased the percentage of apoptotic cells from 2.9% to 40%. The observations made in the study all pointed towards the RSP2 being the peptide candidate that would most likely act by arresting the cell cycle and activating apoptosis pathways, thereby leading to the death of cancer cells. Thus the peptide fraction can be considered a promising source of plant peptides for anticancer therapeutics. It is very likely that RSP2 would regulate the apoptosis process through its influence on the Bcl-2 family and the downstream caspase enzymes that execute the cell death program. The induction of DNA damage evidenced by the comet assay indicates that RSP2 is capable of inducing intrinsic apoptosis through genotoxic stress that is beyond the cell’s repair capacity. The considerable drop in G0/G1-phase cells points to an interruption in the cell cycle’s preparatory phase, which stops the cells from getting to the DNA synthesis (S) phase and thereby committing to division. RSP2, a byproduct of rapeseed processing, is thus a sustainable and economically appealing solution to drug discovery that gives value to agricultural waste [94,95]. Zhai and others (2013) discovered that walnut protein processed with papain contains peptides which could effectively stall the growth of multiple human cancer cell lines with no harmful effects on normal cells. To be more precise, the papain hydrolysate had an IC_50_ of 1.62 mg/mL, 2.04 mg/mL, and 2.21 mg/mL, respectively, for human breast cancer cells (MCF-7), colon carcinoma cells (Caco-2), and cervical carcinoma cells (Hela), where it exhibited anti-proliferative results that were dependent on dosage. Quite importantly, the specific hydrolysate under study did not cause a decline in the population of normal rat small intestinal crypt epithelial cells (IEC-6) and, moreover, even amplified the growth of mouse spleen lymphocytes, which points to its selective cytotoxicity towards cancer cells and possible immune-enhancing properties. The results of this study give an indication that papain hydrolysis will enable the release of specific anticancer peptides from walnut protein that will be able to target malignant cells without doing any harm to healthy tissues, thus proving them to be very effective and functional food components for cancer prevention and therapy [96].

**Figure 8:**
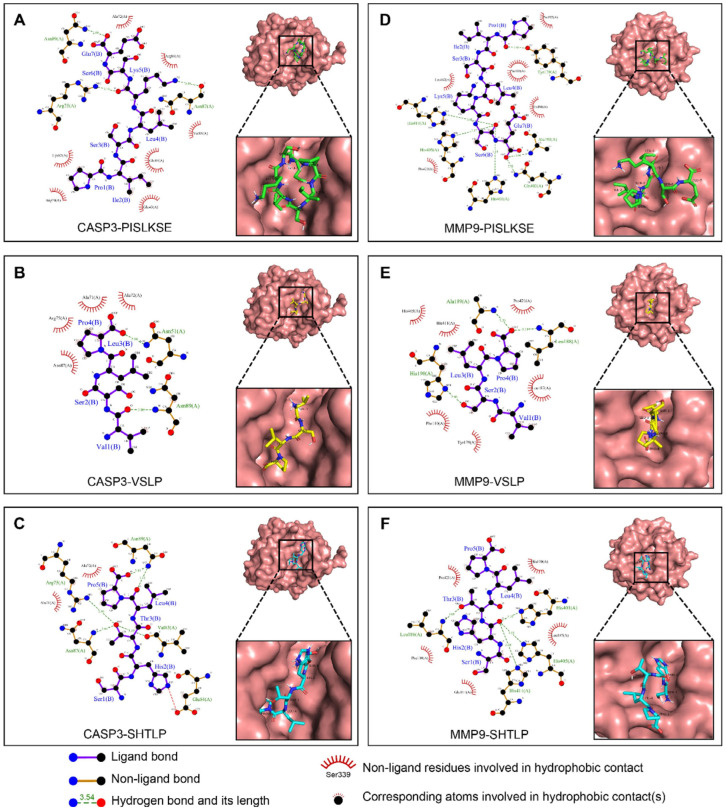
Molecular docking analysis of bioactive peptides PISLKSE, VSLP, and SHTLP with core targets CASP3 and MMP9. (**A**,**C**,**E**) Detailed two-dimensional interaction diagrams depicting the hydrogen bonds (green arrows) and hydrophobic interactions (pink arcs) between each peptide and the caspase-3 (CASP3) active site. (**B**,**D**,**F**) Corresponding interaction diagrams for each peptide bound to the matrix metalloproteinase-9 (MMP9) active site. Binding affinity values (kcal/mol) are indicated for each complex, with more negative values representing stronger binding. The specific amino acid residues involved in the interactions are labelled. Figure is adapted from Xie et al., 2024, Open access article, Journal of Functional Foods [97].

Xie et al. [97] confirmed that the walnut protein hydrolysate WPH-M1 showed wide-spectrum anticancer activity against different types of cancer cells, including HCT116, 769-P, A431, Hep-G2, and A549. The mode of action was through the release of three peptides—PISLKSE, VSLP, and SHTLP—which interacted in a very specific manner with the main cancer cell death (apoptosis) and tissue destruction (MMP9) enzymes. This was further validated by molecular docking studies, which showed that the three peptides bind with very high affinity to both targets (Figure 8). PISLKSE was bound to CASP3 with an affinity of −6.3 kcal/mol. It was found to form hydrogen bonds with Asn89, Asn87, and Arg75, and hydrophobic interactions with Ala72, Arg86, Val85, Glu84, Glu43, Arg79, and Lys82 (Figure 8A). PISLKSE was also found to interact with MMP9 (−5.9 kcal/mol) through hydrogen bonds to Tyr179, Ala191, Gln402, His401, His405, and His411, and hydrophobic contacts with Phe192, Phe110, His190, Pro421, and Leu187 (Figure 8B). VSLP showed a CASP3 affinity of −6.9 kcal/mol, engaging Asn51 and Asn89 through hydrogen bonds and Ala71, Ala72, Arg7, and Asn87 via hydrophobic interactions (Figure 8C). VSLP’s binding to MMP9 (−6.0 kcal/mol) involved hydrogen bonds with Ala189, Leu188, and His190, as well as hydrophobic interactions with His405, His411, Pro421, Leu187, Tyr179, and Phe110 (Figure 8D). SHTLP showed the strongest favorability towards CASP3 (−8.0 kcal/mol), forming multiple hydrogen bonds with Asn89, Val85, Asn87, and Arg75, and hydrophobic contacts with Ala71 and Ala72 (Figure 8E). SHTLP was able to bind effectively to MMP9 (−6.6 kcal/mol) via hydrogen bonds to His401, His405, His411, and Leu188, supported by hydrophobic interactions with Glu111, Phe110, Pro421, His190, and Leu187 (Figure 8F).

These molecular interaction patterns indicate a dual anticancer mechanism: activation of CASP3 to induce cancer cell death, and inhibition of MMP9 to block metastasis [97]. The high binding energy and extensive interaction network of SHTLP suggest that it is a promising candidate for drug development. This computational evidence provides strong structural support for the therapeutic potential of WPH-M1 and highlights the potential of food-derived peptides as sources of novel anticancer agents targeting major regulatory pathways.

He et al. [98] obtained three peptide fractions (PSO1, PSO2, PSO3) from perilla seed protein and tested them against several cancer cells. All three were effective, but their potency varied by cell type. PSO3 worked best against glioma, lung, colon, and liver cancer cells. Against stomach cancer, PSO1 was the strongest. The sequence of PSO3 is Ser-Gly-Pro-Val-Gly-Leu-Trp. Its activity comes from a terminal tryptophan and hydrophobic amino acids (Val, Leu, Trp) that help it enter cells and trigger apoptosis. The central proline may improve stability. The different effects across cell lines suggest that the peptides work through context-dependent mechanisms, possibly mitochondrial apoptosis or blocking survival signals. Another natural peptide, RA-V, is a potent anti-tumor agent. It works by blocking the interaction between two key proteins, PDK1 and AKT, which are part of the PI3K/AKT/mTOR survival pathway. RA-V binds to PDK1 and prevents it from activating AKT. This shuts down the survival signal and triggers the mitochondrial apoptosis pathway, leading to caspase activation and cell death. Experiments showed that combining RA-V with a PI3K inhibitor increased its effect, while an overactive AKT mutant reduced it. Thus, RA-V selectively kills cancer cells by turning off AKT-driven survival signals and activating mitochondrial apoptosis [99].

Peptide fractions AGP and ABP, sourced from Abrus lectins, were found to have strong immunostimulatory effects both in vitro and in vivo on Dalton’s lymphoma (DL)-bearing mice, thereby proving their potential as new cancer immunotherapy agents. The activation of splenocytes by both peptides was very effective, while in addition they polarized the adaptive immune system into a strong Th1-type immune response, which is very important for anti-tumor immunity, characterized by the increased secretion of the main cytokines as IL-2, which drives T-cell proliferation, IFN-γ, which activates macrophages and enhances antigen presentation, and TNF-α, which can induce direct apoptosis in sensitive tumor cells. Flow cytometry, performed in a comprehensive way, revealed a major increase in the percentage of both CD3+ T lymphocytes and CD19+ B cells, which demonstrated a highly activated phenotype by the high surface expression of the activation markers CD25 and CD71, besides the fact that the levels of the co-stimulatory CD80 and CD86 molecules on antigen-presenting cells were significantly higher compared to the tumor control, indicating a shift from immune tolerance to effective activation. The therapy not only remarkably but also significantly improved the anti-tumor capability of the tumor-associated macrophages, transforming them from a pro-tumor M2 type to an anti-tumor M1 type through nitric oxide production stimulation—a crucial cytotoxic mediator—an increase in pro-inflammatory cytokine IL-1 secretion, and high phagocytic activity against tumor cells; on the other hand, it reduced the expression of the characteristic M2 marker mannose receptors. In addition to that, it was found that both AGP and ABP could stimulate Natural Killer (NK) cells directly, thus boosting their innate capacity to identify and destroy MHC-deficient tumor cells through perforin and granzyme release. Taken together, these complex findings suggest that AGP as well as ABP are powerful and effective immunomodulators that trigger a concerted tumor attack by means of a simultaneous increase in both innate and adaptive immune mechanisms, thus restoring immuno-surveillance and creating an unfavorable microenvironment for cancer to grow [100]. A patent of Jilin Guorui Pharmaceutical Co., Ltd. (Huainan, China) reveals a new anti-angiogenic and anti-cancer peptide Lyc-Arg-Trp-Cys-Phe-Leu-Pro, and it is a Codonopsis lanceolata-derived one. The researcher prepared the peptide by managing an oligopeptide mixture and identifying it via HPLC-MS/MS, and then again purified it through Sephadex LH-20 column chromatography and HPLC. The peptide has been verified to possess very good anti-angiogenesis activity and to stop the growth of cervical cancer; hence, this food-originating active peptide could be applied in functional foods, health care products and pharmaceuticals for the prevention and treatment of cervical cancer-related diseases [101].

The anti-tumor effects of corn-derived peptides (CPs) are of great significance. They selectively trigger apoptosis in HepG2 human hepatocellular carcinoma cells signaling this way through the strict regulated intrinsic mitochondrial pathway, which is the main checkpoint for the removal of cancer cells. The main process of apoptosis here is controlled by changes in the Bcl-2 family of proteins, especially an increased Bax/Bcl-2 ratio, which leads to the formation of pores in the outer mitochondrial membrane (MOMP). This is happening alongside the transcriptional activation and phosphorylation of the key tumor suppressor protein p53, which finally results in the proteolytic cleavage and enzyme activation of the main executioner protein caspase-3. All the above processes eventually take the cell to an irreversible state of biochemical and morphological death changes, as in the case of programmed cell death. Also, CPs not only have direct toxic effects but also significantly increase the innate and adaptive anti-tumor immune response of the host within the tumor microenvironment and systemically adapt it by an increase in the serum and splenic levels of key immunostimulatory cytokines such as IL-2, which is the main driving force behind T-cell proliferation and differentiation, IFN-γ, which is responsible for macrophage activation and increasing its ability to present antigens, and TNF-α, which has the ability to cause tumor cell death directly, thus leading to the activation of inflammatory responses. Hepatocellular carcinoma (HCC) growth and proliferation in vivo were thus markedly reduced, survival was improved, and there was a significantly lower tumor burden in mice models [102] as a consequence of these two strong and mutually enhancing mechanisms: the direct pro-apoptotic effect on cancer cells and the wide systemic immunomodulation that activates cytotoxic T-lymphocytes and neutralizes immunosuppressive factors together and strongly [103].

Novel cationic nonapeptide, the primary structure of which is Ala-Trp-Lys-Leu-Phe-Asp-Asp-Gly-Val, was successfully isolated and purified from the seeds of sago-palm, Cycas revoluta. This peptide displays a variety of significant biological activities, with the most notable being very effective against cancer. The mode of action is direct and involves the binding of the peptide to the cellular DNA with high affinity. This specific binding takes place through electrostatic attractions where the positively charged lysine residue (Lys3) is attracted to the negatively charged DNA phosphate backbone, and through a network of specific hydrogen bonds mainly involving the N-terminal Ala1 residue and the aspartic acid residues (Asp6 and Asp7). Such molecular interaction has cellular-level consequences. The peptide intercalation disrupts the nucleosome structure, DNA is unwound, and replication and transcription, which are essential processes, are hindered. A cytotoxic peptide was extracted from the tryptic hydrolysate of Polyalthia longifolia seed proteins and shown to be very effective in cancer treatment. The peptide fraction known as F2 caused strong toxicity in A549 lung cancer and HeLa cervical cancer cells at very low concentrations of 10 µg/mL and 30 µg/mL, respectively. The apoptotic nature of the mechanism of action was confirmed by DNA fragmentation and an increase in sub-G0 phase cells in both cell lines. Chemical structural studies showed high purity (>90%) and a molecular weight of about 679.8 Da. Thus, the authors have pointed out the use of F2 as a potent apoptotic inducer with potential therapeutic applications in cancer treatment [104].

**Figure 9:**
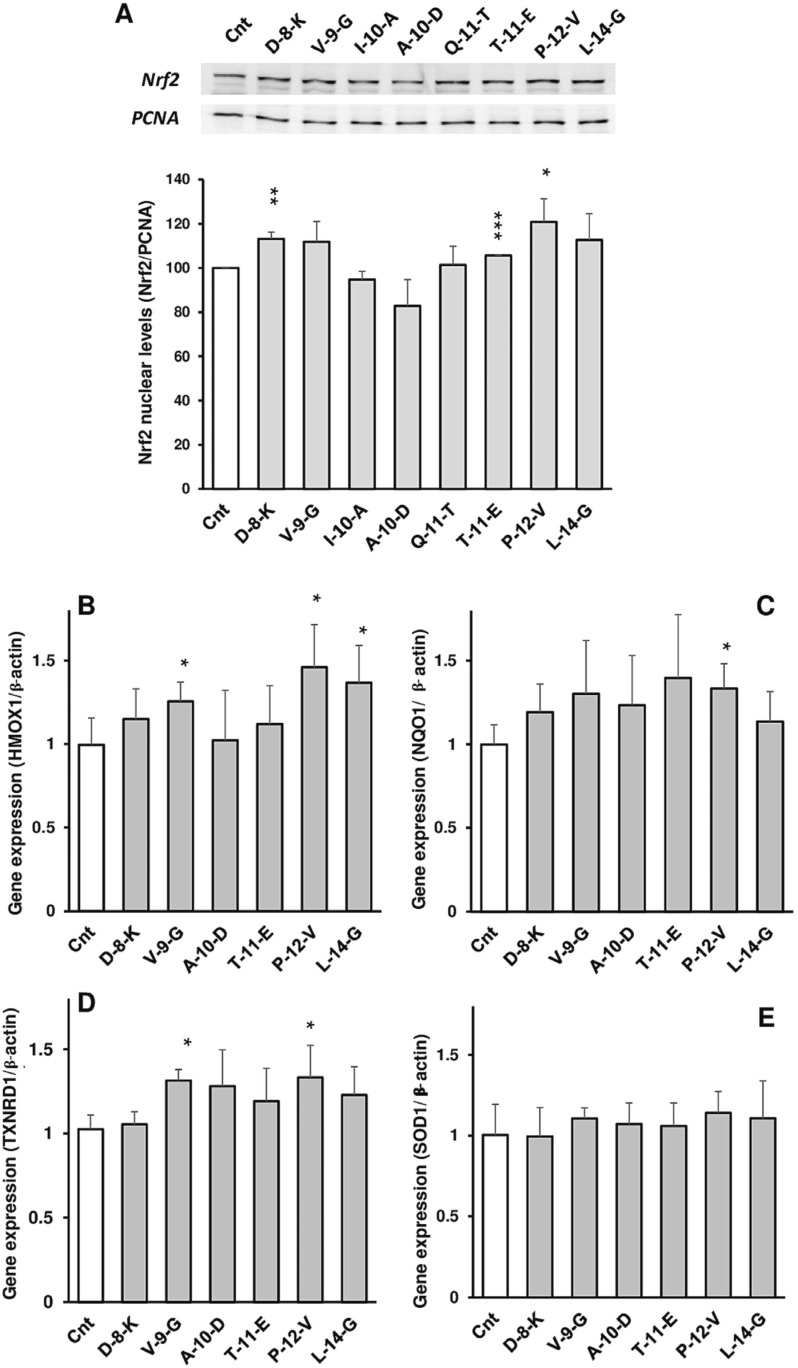
Activation of the Keap1/Nrf2 Pathway and upregulation of antioxidant enzymes in caco-2 cells treated with sunflower-derived peptides. (**A**) Nuclear translocation of Nrf2 was assessed by Western blot analysis of Nrf2 protein levels in nuclear fractions isolated from Caco-2 cells following treatment with the indicated peptides. A representative blot is shown. The accompanying bar graph presents the densitometric quantification of Nrf2 levels from three independent experiments, normalized to the loading control PCNA (Proliferating Cell Nuclear Antigen). (**B**–**E**) mRNA expression levels of key antioxidant enzymes regulated by the Antioxidant Response Element (ARE) were measured by quantitative real-time PCR (qRT-PCR). Caco-2 cells were treated with the indicated peptides, and the expression of (**B**) HMOX1 (Heme Oxygenase-1), (**C**) NQO1 (NAD(P)H Quinone Dehydrogenase 1), (**D**) TXNRD1 (Thioredoxin Reductase 1), and (**E**) SOD1 (Superoxide Dismutase 1) was analyzed. Figure is adapted from Tonolo et al., 2024, Open access article, Elsevier Open access, Food chemistry [105]. *** *p* < 0.001, ** *p* < 0.01, * *p* < 0.05.

The Sunflower Peptide-Enriched Fraction (PEF) together, with the constituent peptides, have been shown to have an anticancer-related effect via the antioxidant, anti-inflammatory and metabolic pathways, rather than through direct cytotoxicity in Caco-2 cells. The anticancer property is mostly directed by the activation of the Keap1/Nrf2 antioxidant pathway (Figure 9A). Through molecular docking, the peptides D-8-K, T-11-E, and P-12-V were recognized as the strongest binders to Keap1, thus inducing Nrf2 translocation into the nucleus. Such activation led to the up-regulation of the expression of the antioxidant enzymes HMOX1, NQO1, and TXNRD1, thereby boosting the cellular defense against oxidative stress (Figure 9B–D). Nonetheless, the up-regulation of SOD1 expression through Nrf2 activation by the peptides was not as marked (Figure 9E). The peptides have also been shown to be effective in the reduction of ROS production and lipid peroxidation caused by the pro-oxidants like TbOOH, thereby allowing the cells to escape from the oxidative damage—the main culprit of cellular dysfunction and cancer progression. Peptide P-12-V, in particular, showed its anti-inflammatory activities through the prohibition of the nuclear translocation of NF-κB and the downregulation of pro-inflammatory cytokines (IL-6, IL-8, and TNF-α), which are usually found in the cancer microenvironment at high levels and are responsible for the survival and growth of the tumors. Moreover, certain peptides were implicated in the regulation of mitochondrial metabolism, which is in line with their protective action. The peptides P-12-V, D-8-K, and V-9-G all produced a decrease in both the basal and ATP-linked oxygen consumption rates (OCR), thus revealing a change in mitochondrial activity. It is known that mitochondrial respiration, when reduced, can lead to a decrease in the generation of ROS; hence this metabolic alteration can be seen as another mechanism of reducing oxidative stress and thereby creating an easier condition for the cancer to grow [105].

The peptide fraction from moth bean (*Vigna aconitifolia* (Jacq.) MBP), which is formed by the enzymatic hydrolysis of seed protein by alcalase, shows strong anticancer, antioxidant, and DNA-protective activities. Among the many beneficial effects, MBP was a very effective antioxidant, since it managed to eliminate DPPH and ABTS radicals and showed powerful metal chelating ability. Additionally, it was responsible for MCF-7 breast cancer cell death with a very high degree of strength. In addition, the peptide fraction was a protector against DNA damage; thus, it was capable of reducing the genetic instability caused by oxidative stress. The peptide fraction was a protector against DNA damage and was thus capable of reducing the genetic instability caused by oxidative stress. These multifunctional features of MBP make it a strong candidate for application as a bioactive agent in the nutraceutical or therapeutic domain for cancer and oxidative stress-related damage prevention and treatment [106].

The anticancer activity of cowpea peptides is powerful enough to place them at the top of the list of potential therapeutic candidates. The peptides, in fact, are capable of bringing about the inhibition of cancer cells and of causing their death by apoptosis through the mechanisms of oxidative stress induction and mitochondrial pathway activation. Their anti-inflammatory properties not only help to alleviate the tumor effects by acting on the tumor microenvironment but also enhance their anticancer effects. In addition, there is some evidence that cowpea peptides can interact with polyphenols and thus boost their overall bioactivity not just against cancer but also against diabetes and heart diseases. All these multifunctional benefits value cowpea-derived peptides for their use in nutraceutical and pharmaceutical applications for cancer prevention and treatment [107].

The enzymatic breakdown of soybean 7S protein produces the antioxidant pentapeptide Leu-Leu-Pro-His-His. The tripeptide Arg-Gly-Asp (RGD) has also been characterized as the principal adhesion site of soy lunasin. This sequence is necessary for lunasin’s interaction with cells. The interaction with cells is, in turn, the prerequisite for the anticarcinogenic properties of lunasin [108,109]. Apart from lunasin, soy protein also consists of some cancer preventing peptides. One such peptide is an anticancer nonapeptide whose molecular weight is 1157 Da and whose sequence is X-Met-Leu-Pro-Ser-Tyr-Ser-Pro-Tyr. This peptide was isolated from defatted soy protein by a complicated multi-step process that included thermoase hydrolysis, ethanol extraction, and sequential chromatography with XAD-2 hydrophobic resin, Sephadex G-25, and C18 HPLC. The purified peptide was found to be very toxic to P388D1 mouse monocyte macrophage cells with an IC_50_ value of 0.16 mg/mL. The anticancer action was demonstrated by the inhibition of cell cycle progression, and the arrest at the G2/M phases was induced when the concentration was 1 mg/mL, thus hindering cellular expansion and leading to the death of cancer cells [110]. In their study, Hsieh et al. [111] found that hydrolytic peptides from soy protein (SB and ST) had a pronounced anticancer effect on human oral squamous carcinoma cells (HSC-3). Both peptides were capable of inhibiting the proliferation of cancer cells in a time- and dose-dependent manner, with respective IC_50_ values of 0.74 mg/mL for SB and 0.60 mg/mL for ST after 72 h of treatment. The lack of cytotoxic effects on normal human oral keratinocytes (NHOK) cells means that the peptides have selective toxicity against cancer cells. Blocking the cell cycle progression at the S-phase was one of the modes of action that resulted in the downregulation of cyclin E, cyclin A, and CDK2, along with no effect on p21 and p27 protein expression. Additionally, both of the hydrolysates initiated apoptosis in HSC-3 cells, which was evidenced by mitochondrial depolarization and higher annexin V staining. ST was further shown to specifically trigger apoptosis through the mitochondrial pathway, illustrated by the downregulation of Bcl-2, PARP, caspase-3, and caspase-9, and the upregulation of p53 and cleaved caspase-3. Conversely, SB seemed to cause necrosis at a concentration of 1 mg/mL, as determined by the increase in propidium iodide-positive cells along with the absence of classic apoptotic markers [111]. Marcela et al. [112] conducted research on the three peptide fractions derived from six-day germinated soybean protein hydrolysis (>10 kDa, 5–10 kDa, and <5 kDa) and evaluated their antioxidant and anticancer properties. Among the fractions, the one greater than 10 kDa was noted for its superior antioxidant power and even showed considerable inhibition of the cell proliferation of the HeLa, SiHa, CasKi (cervical) and MCF-7, MDA-MB-231 (breast) cancer cell lines. The most active peptide fraction (MAPF) was determined to have IC_50_ values of 16.2, 14.3, and 15.2 mg/mL against HeLa, CasKi, and MDA-MB-231 cells, respectively, and it was capable of inducing apoptosis with indices over 50% in the course of 6 to 8 h. Notably, MAPF posed practically no toxicity to the normal HaCaT cells. The active site of the peptide was claimed to be a result of the amino acids proline, phenylalanine, and tyrosine being in high proportions, and the extent of the size of the peptides present was from 12 to 42 kDa [112]. Chen et al. [113] succeeded in isolating and identifying a new antioxidant and anticancer peptide gourd from black soybean byproducts. They managed to obtain a peptide fraction (F2-c) with a molecular weight of 455.0 Da and the sequence Leu/Ile-Val-Pro-Lys (L/I-VPK) through bioactivity-guided purification comprising ultrafiltration, gel filtration and RP-HPLC. This peptide showed an extremely strong radical scavenging ability with an IC_50_ value of 0.12 µM (DPPH) and 0.037 µM (OH•). It also possessed strong cytotoxicity against cancer cell lines HepG2 (0.22 µM), MCF-7 (0.15 µM), and HeLa (0.32 µM). Molecular docking showed that the peptide binds very well to key proteins related to apoptosis, particularly caspase-3, with hydrophobic interactions and hydrogen bonds, indicating a possible mechanism of cell death promotion. The peptide was considered a promising bioactive agent for the development of functional food or anticancer therapeutics [113].

The research by Quintal Bojórquez et al. [114] revealed the presence of three new peptides—KLKKNL, MLKSKR, and KKYRVF—obtained from the seeds of Salvia hispanica (chia), which were selectively cytotoxic against certain human cancer cells. Among the three, the peptide KLKKNL was the most potent in causing apoptosis in Caco2 and HeLa cells, whereas KKYRVF caused the strongest selective cytotoxicity effect across MCF-7, Caco2, HepG2, and DU145 lines by inducing necrosis. The results reveal the possibility of using peptides derived from chia as selective anticancer agents, since they act through different mechanisms (apoptosis vs. necrosis), depending on the type of peptide and cancer cell used [114]. Peptides obtained from the waste of chickpea (CWP) and pea (PWP) seeds showed a significant anticancer effect. CWP strongly inhibited HePG-2 and MCF-7 cancer cell lines, with an 83–86% inhibition at 100 µg/mL and an IC_50_ of 20 µg/mL. PWP also had considerable anticancer activity, but it was less potent, having an IC_50_ of 25 µg/mL. These findings encourage the idea of using peptides from legume seed waste as natural chemopreventive agents due to their selective cytotoxicity toward cancer cells [115].

Among the different fractions of purified proteins derived from the seeds of Chenopodium quinoa, the one at pH 2 showed the most potent anticancer activity against both A549 and HeLa cell lines. This particular fraction also gave the maximum proteolytic activity (2.451 units/mL), implying that there might be a connection between its enzymatic activity and its cytotoxicity. Using LC-MS/MS, it has been suggested that peptides present in this fraction are responsible for its anticancer effect; however, precise sequences and mechanisms (for instance, apoptosis induction, cell cycle arrest) still need to be elucidated. Altogether, these results have put forward peptides from quinoa as potential candidates for the future development of either therapeutics or nutraceuticals that specifically target cancer cells [116].

Peptide hydrolysate obtained from corn gluten meal (CP) consists mostly of short-chain di- and tripeptides as a by-product of the proteolytic process with alkaline protease from Alkalophilic Bacillus A-7. The use of an in vivo model of DMBA-induced mammary carcinogenesis in female Sprague-Dawley rats (starting at 35 days of age) allowed for the demonstration of tumor progression inhibition by a 10% CP–peptide mixture in the diet, which resulted in a decrease in both tumor incidence and burden. The primary anticancer mechanism considered ruled out the hormonal pathway since serum 17β-estradiol levels did not differ among the control (CAS) and treatment groups. Importantly, the protective effect was especially related to the structure of the bioactive peptide, as a simulated free amino acid (AA) mixture of CP did not display the full efficacy, indicating that the short-chain peptides’ intrinsic properties rather than their general nutritive value are involved in the mechanism [117]. In vitro and in vivo studies by Moritani et al. [118] demonstrated the clear beneficial effect on cell death induced by oxidative stress, as the oryza peptide OP60 was the one positively influencing the results. In HepG2 cells, the application of OP60 before the exposure to hydrogen peroxide and acetaminophen showed a protective effect that depended on the cell count. This was measured by less LDH being released and the intracellular reduction in redox homeostasis being preserved through the total glutathione (GSH) levels and the GSH/GSSG ratio restoration. The rescue of the cells was through the activation of Nrf2, as the OP60 treatment increased Nrf2 mRNA, total and nuclear Nrf2 protein levels, and the high Nrf2-regulated antioxidant enzymes like the γ-GCS subunits (important for GSH synthesis), HO-1, NQO1, and GR increased. The criticality of Nrf2 was supported by the use of siRNA knockdown, which stopped the rising of GSH levels and the expression of γ-GCSh and HO-1 that were previously enhanced by OP60. The same pharmacological properties that displayed the same pharmacological properties during the administration of OP60 in mice in an acetaminophen-induced liver injury assay demonstrated a marked decrease in liver injury biomarkers (AST, ALT, LDH, ALP) in serum, less penetrating histopathological damage and the restoration of liver GSH levels. Moreover, APAP-treated mice livers with OP60 pretreatment exhibited a strikingly higher γ-GCSh and HO-1 protein expression, confirming that its mechanism of action in vivo indeed operates through the reinforcement of the antioxidant defense system via Nrf2 pathway activation, leading to enhanced GSH production and cell death protection [118]. Yang et al. [119] has shown that industrially produced rice protein peptides (RPP) have a large effect on reducing the symptoms of acute colitis in mice, induced by the application of dextran sulfate sodium (DSS). The treatment with RPP reduced the primary disease parameters such as weight loss, colon shortening, and tissue damage; hence the disease activity index (DAI) decreased. The protective mechanism consisted of two aspects: RPP firstly balanced the inflammatory cytokines and oxidative stress by triggering the Keap1-Nrf2 signaling pathway, which ultimately resulted in the upregulation of antioxidant enzyme expression and increased the production of intestinal tight junction proteins to fortify the barrier function. Secondly, RPP had a prebiotic effect through gut microbiota modulation, especially by raising the Firmicutes/Bacteroidetes (F/B) ratio, increasing the relative numbers of beneficial bacteria like Akkermansia, and controlling the amounts of short-chain fatty acids. The authors concluded that RPP is effective against colitis via these intertwined mechanisms of activating the Keap1-Nrf2 antioxidant pathway and reestablishing healthy gut microbiota, which brings into light its possible application as an effective dietary supplement or functional food in the management of inflammatory bowel disease [119].

Corn peptides (CPs), a product of the enzymatic hydrolysis of corn gluten meal, exhibit strong and varied anti-tumor actions against hepatocellular carcinoma (HCC) via combined direct cytotoxic and indirect immunomodulatory mechanisms. In laboratory tests, CPs cause human liver cancer HepG2 cells to die by first stopping their division and then activating the internal mitochondrial pathway; the latter is marked by a noticeable increase in the Bax/Bcl-2 ratio, activation of the tumor suppressor p53, and the cutting of executioner caspases like caspase-3, all of which lead to programmed cancer cell death. In experiments on mice with H22 tumors, CPs not only directly induced apoptosis but also anticancer effects by significantly boosting the immune system of the host; the latter was quantitatively evidenced by a rise in spleen index, i.e., the activation and proliferation of lymphocytes, and by the higher blood levels of important cytokines such as IL-2, IFN-γ, and TNF-α, which make cytotoxic T-cell and NK cell activity stronger, thereby fortifying cell-mediated immunity and creating a hostile microenvironment for tumors. The merging of these two mechanisms led to a powerful overall antitumor effect, which was eventually demonstrated by a considerable decrease in tumor volume and mass, and most significantly, by a considerable prolongation of the survival period of the treated mice, thereby indicating the potential of CPs as a natural and safe bioactive agent that could be used in the management of hepatocellular carcinoma as either an adjuvant therapeutic or preventive functional food. Corn peptides (CPs) have shown impressive anti-cancer properties based on two different mechanisms of action. The results from in vitro studies indicated that the CPs were able to cause cell death in human hepatoma HepG2 cells by preventing the cells from advancing through the cell cycle, thereby increasing the Bax/Bcl-2 ratio, along with the activation of p53 and Cleaved-caspase-3 as the key proteins involved. On the other hand, in vivo studies indicated that CPs resulted in a considerable decrease in the size of tumors in H22 tumor-bearing mice. The efficacy in this case was not only due to the CPs causing the death of the cancer cells directly but also due to the CPs boosting the immune system of the host to a noticeable level, as indicated by the increase in the spleen index and the increase in the levels of the cytokines IL-2, IFN-γ, and TNF-α. Moreover, CPs prolonged the survival time of the mice, which points to the potential of using them as a safe and efficient bioactive agent or functional food against hepatocellular carcinoma [120]. The anti-cancer potential of a peptide fraction derived from the hydrolysate of germinated soybean protein in HeLa cervical cancer cells has been demonstrated. The proteins were extracted from soybeans that had been germinated for six days, and after the removal of ethanol-soluble phytochemicals, they were subjected to hydrolysis by digestive enzymes. Ultrafiltration was then used to separate the hydrolysate into five different peptide fractions based on their respective molecular sizes. The fraction that had the highest biological activity (>10 kDa) was notably the only one that has been able to cause a dramatic reduction in cancer cell viability. The method through which it did so has been uncovered to be apoptosis, which was further corroborated by the activation of the caspase cascade and DNA fragmentation. On top of that, this strong peptide fraction was also shown to reduce the expression of two key cancer genes, PTTG1 and TOP2A, at the mRNA level, which are already recognized therapeutic targets. The authors of the study confirm that soy protein isolates from the germinated seeds are a potential source of peptides for cancer preventive or therapeutic functional food application [121]. Leguminous Limyin peptide (6.8 kDa) has strong and specific cytotoxic activity that completely kills liver hepatoma Bel-7402 and neuroblastoma SHSY5Y tumor cell lines. Its mechanism of action is through inducing apoptosis and arresting the cell cycle of cancerous cells. Interestingly, its cytotoxicity is selective, usually leaving normal cells unharmed, and it can withstand high temperatures. Such a powerful combination of selecting the right type of cancer cells and an impressive living trait makes the defensin-like peptides in legumes very promising as new therapeutic agents or functional food components for cancer management [122].

Wang and Ng explored a peptide of 7.3 kDa molecular weight, which was obtained from the common bean (Phaseolus vulgaris cv. “Spotted bean”) and has considerable similarity with plant defensins. The peptide had a very strong antiproliferative effect on both leukemia (L1210) and lymphoma (MBL2) cell lines. In a 2011 study, a different source reported that a dimeric hemagglutinin from the same source had been successfully used to treat breast cancer cells (MCF-7) with an IC_50_ of only 0.2 μM. The hemagglutinin led to cell death through the death receptor-mediated pathway, which was demonstrated by a combination of G2/M phase cell cycle arrest, phosphatidylserine externalization, and mitochondrial membrane depolarization. The apoptotic cascade that followed included the up-regulation of Fas ligands, caspase-8, and caspase-9, processing of BID and Lamin A/C, and the release of p53 [123,124].

Peptides isolated from the hydrolysis of albumin of white hybrid and quality protein maize have shown a strong inhibitory effect on the proliferation of human HepG2 liver cancer cells. The major mechanism by which the peptides exert this effect is apoptosis, which is the natural death of cells. The individual pure peptides did not show such pro-apoptotic activity, but the complex peptide fractions were the only ones that did. The fractions were able to do this by downregulating the major antiapoptotic proteins in the cancer cells, thus making the balance of the cells tipped towards death. The fraction that was extracted from the white hybrid maize (Asgrow-773) was the one that showed the highest potency, thus indicating the dependence of bioactivity on the genotype. Moreover, this implies that the albumin peptides from maize also have a multi-target mechanism that inhibits the growth of cancer cells [125]. The peptide from Nepalese large red beans was found to have a considerable effect in inhibiting cancer cell proliferation. Its IC_50_ was established at 15 µM for L1210 murine leukemia cells, which means it could effectively stop the growth of these cells. It also showed activity against MBL2 lymphoma cells, where it demonstrated an IC_50_ of 60 µM. Therefore, these results indicate the very strong, dose-dependent inhibition of cell proliferation. The suppression was more pronounced in leukemia cells than in lymphoma cells, as indicated by the lower IC_50_ for leukemia cells [126].

Pyrularia thionin, which has been obtained from *Pyrularia pubera*, shows marked potential as an antitumor agent due to its strong cytotoxic properties. The compound was found to be active at the level of HeLa and B16 cell lines with an IC_50_ value of 50 μg/mL. Nevertheless, at the same time, this anticancer action is totally dependent on a very toxic mechanism. Cytotoxicity is the consequence of the disruption of the cell membrane, leading to its loss of polarization and opening the way for calcium (Ca^2+^) ions to enter the cell through other channels. The influx of calcium ions, in turn, causes the activation of an intracellular phospholipase A2 that leads to the loss of membrane integrity and, consequently, to cell death. The important contribution of particular molecular features to the toxicity process is pointed out by the observation that the iodination of the thionin’s tyrosine residues greatly diminishes its toxic effects [127].

Kong et al. [128] have presented the data of a new polypeptide called viscotoxin B2 that was obtained from *Viscum coloratum* and whose primary structure is KSCCKNTTGRNIYNTCRFAGGSRERCAKLSGCKIISASTCPSDYPK. Viscoxin B2 breeds a high sequence resemblance to known thionins from *Viscum album*, hence being classified as a plant thionin, a group of proteins that are famous for their cytotoxicity. Pharmacological studies showed that viscotoxin B2 has a unique cytotoxicity towards tumor cells, showing significant anticancer activity against rat osteoblast-like sarcoma with a strong IC_50_ value of 1.6 mg/L. This efficacy means that it is a potentially important compound in the area of cancer therapy drug development [128]. The three-dimensional NMR structure of Viscontoxin C1 suggests that has the conserved helix-turn-helix and β-sheet fold typical of thionins. The similarity in structure is so high that one might mistake it for other α- and β-thionins known for antimicrobial effects, but the viscotoxin subfamily is characterized by its powerful and specific cytotoxicity against tumor cells, e.g., rat sarcoma, which is a reason for the pronounced anticancer activity. Its unique electrostatic surface properties are likely the reason for its selective targeting and disruption of cancer cell membranes [129]. Viscotoxin A3 (VA3) is considered a selective and tumor-specific treatment, as it binds to phosphatidylserine (PS) lipids the way a normal cell would because PS is the only layer that is most exposed on the membrane of tumor cells. The exposure is mostly 7–8 times greater in tumor cells than in non-tumor cells, thus creating a very specific point for VA3 to attach and change the permeability of the membrane. It is thought that the toxin groups the lipids together, thus leading to the attraction of more lipids, i.e., severe membrane perturbations, which finally drive the cell to death. This is the reason why VA3 has a different toxicity towards the tumor cells than to normal ones, such as erythrocytes [130,131].

In a paper by Li et al. [132] the authors mentioned finding a new, cytotoxic protein Ligatoxin B and class 3 thionin from the mistletoe *Phoradendron liga*. The amino acid sequence of the protein was identified as a chain of 46-amino acids: 1-KSCCPSTTAR-NIYNTCRLTG-ASRSVCASLS-GCKIISGSTC-DSGWNH-46. This thionin proved to be very effective in vitro against a variety of human cancer cell lines, among which was the resistant renal adenocarcinoma ACHN that required the lowest concentration of the drug (IC_50_ = 3.2 µM), as well as the lymphoma U-937-GTB (IC_50_ = 1.8 µM). When discussing possible mechanism of action, the researchers put forward a highly original and very important theory which was backed up by the results of their structural studies. They pointed out that Ligatoxin B has thionins’ defining three-dimensional architecture, which is based on a helix-turn-helix and a short antiparallel β-sheet. What is more, they were able to show that the helix-turn-helix region is very much like the DNA-binding regions found in some HTH proteins, for instance, the lambda repressor. This structural evidence made them think that the cytotoxicity of thionins, such as Ligatoxin B, might not only be caused by membrane fragmentation but also by their attachment to the DNA in the cell. This possible DNA-binding activity is mentioned as a possible mechanism for bypassing the resistance of cancer cells to drugs and marks a new path in the study of the essential role of thionins in the physiological processes of plants [132].

Different thionins, phoratoxins A to F, have been found in the *Phoradendron tomentosum*, each of which is toxic. Phoratoxins A and B are extremely poisonous to rats (LD_50_ 0.5–1 mg/kg) and heart function alteration is the main cause of their toxicity. Moreover, phoratoxins C-F are able to cause significant and varied anticancer activities on solid and hematological tumor cell lines. Phoratoxin C was the most potent, with a IC_50_ of 0.16 µM, while phoratoxin F was the least potent, with an IC_50_ of 0.40 µM. Moreover, phoratoxin C killed the primary patient-derived cells selectively and was thus 18 times more effective against breast cancer cells from solid tumors than against hematological cancer cells. This makes phoratoxins a promising alternative for new anticancer agents, especially for solid tumors. The ability of these peptides to act differently is remarkable, but their genetic sequence shows over 90% similarity. This indicates that tiny shifts in the particular amino acids are probably the principal reason behind their different biological activities [133,134]. The Thi2.1 thionin of Arabidopsis thaliana is yet another thionin with anticancer effects. A study performed using a heterologous expression system indicated that its conditioned media suppressed the growth of MCF-7, A549, and HeLa cells by 94%, 29%, and 38%, respectively. A significant disadvantage is its strong cytotoxicity to normal bovine cells, leading to 89% and 93% kill rates in the respective mammary epithelial and endothelial cells. The mode of action for Thi2.1 remains unknown [135]. The anticancer activity of Plant Thionins is shown in Table 1.

Plant defensins are a category of antimicrobial peptides which not only possess but also show strong anticancer and cytotoxic effects. The first plant defensin with documented anticancer effects was sesquin, obtained from *Vigna sesquipedalis*, which could even stop the growth of MCF-7 breast cancer and leukemia M1 cells at a concentration of 2.5 mg/mL. This finding opened up the exploration of plant defensins as a source of new anticancer agents [136]. Lunatusin, a defensin from Chinese lima bean (*Phaseolus lunatus* L.) seeds, is credited with MCF-7 breast cancer cells’ growth inhibition at the concentration of 5.71 µM (IC_50_). The antibacterial, human cell translation-inhibitory activity in rabbit reticulocyte lysates is also claimed by it. Thus, lunatusin cannot be said to be selectively toxic to cancer cells and may even cause death in normal tissues and other cell types, leading to its limited usage in therapy. Nevertheless, it still holds a strong position in the research of structure–activity relationships [137]. A defensin-like peptide of 6.5 kDa was isolated from the seeds of the shelf bean through a multi-step purification process and was termed limenin. This peptide was found to have direct anticancer activity via DNA synthesis inhibition. It showed a marked reduction in [methyl-^3^H]-thymidine uptake in M1 myeloma and L1210 leukemia cells, hence having the ability to suppress proliferation in these cell lines. This particular inhibition of cancer cell replication brings about the possibility of using the same as a therapy for the treatment of hematological cancers [138]. From purple pole beans (Phaseolus vulgaris), a defensin peptide of 5443 Da was purified. This peptide was able to kill tumor cells at a great rate while being selective, as it killed only some types of human cancer cells and not normal embryonic liver (WRL68) cells. It was very potent against HepG2, MCF7, HT29, and SiHa cells, with an IC_50_ value of 4.1 ± 0.8 µM, leading to its classification among other plant defensins’ potency. The peptide’s selective cytotoxicity towards normal cells and its malignant counterparts emphasizes its potential as a promising anticancer agent [139]. Coccinin, a 7-kDa defensin-like peptide from small scarlet runner beans (*Phaseolus coccineus* cv. ‘Major’), was found to be very effective against HL60 and L1210 leukemia cells, with an IC_50_ varying from 30 to 40 µM. Still, it displayed no effect on the growth of normal mouse spleen cells; therefore, it was considered to be selectively toxic to tumor cells and non-immune cells [140]. The 5422 Da defensin phaseococcin isolated from *Phaseolus coccineus* cv. ‘Minor’ showed selective anticancer activity. It inhibited the proliferation of HL60 and L1210 leukemia cells with an IC_50_ of 30–40 µM. Importantly, the peptide exhibited no cytotoxic effect on the proliferation of normal mouse splenocytes or on protein synthesis. This selective toxicity highlights its potential as a promising candidate for anticancer development, as it targets malignant cells while sparing normal immune cells [141]. The media conditioned by the bovine endothelial cells that harbor the cDNA of the Capsicum chinense defensin γ-thionin were able to totally abolish the viability of HeLa cervical cancer cells, displayed by 100% inhibition. In addition, this cytotoxic effect was selective as the media had no impact on the viability of the immortalized bovine endothelial cells [142].

**Figure 10:**
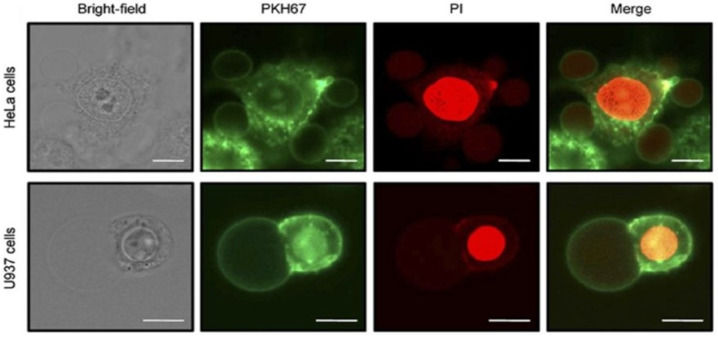
NaD1 induces cell death in HeLa cells through membrane binding, permeabilization, and lysis. Bright-field images capture the sequential morphological changes in HeLa cells following NaD1 treatment, including initial membrane blebbing that progresses to cell swelling and eventual rupture. The pre-labelling of the plasma membrane with the green fluorescent dye PKH67 reveals severe disruption of membrane integrity, indicated by a distorted and fragmented signal. The influx of propidium iodide (PI), evidenced by intense red nuclear fluorescence, confirms the loss of membrane integrity and allows for the visualization of cell death. The merged image demonstrates the direct association between the sites of membrane disruption (green) and the entry of the viability dye (red), correlating with the morphological changes that culminate in complete lysis. Figure is adapted from Poon et al., 2014, Open access article, Elife [143].

The protein NaD1, which comes from plant sources, has anticancer action via a direct adhesion mechanism to the plasma membrane of cancer cells, namely lytic, and is seen in the cancer cell lines HeLa (cervical carcinoma), U937 (monocytic lymphoma), and PC3 (prostate cancer) (Figure 10). This direct binding results in quick membrane protrusions and is very much dependent on PIP_2_ being the lipid that interacts most with NaD1, the reason being the delayed permeabilization of the HeLa cells that have the PIP_2_-sequestering probe GFP-PH(PLCδ) expressed. The permeabilization happens in a very localized manner at the spots where blebbing occurs, allowing the entrance of small molecules like propidium iodide and even of 4-kDa FITC-dextran. The detection through fluorescence shows that NaD1 has been accumulated on the plasma membrane and on the intracellular organelles, indicating that there is a more extensive disruption of the membrane. The whole process ends with total cell death, which has been proven by the release of lactate dehydrogenase (LDH), and depending critically on the particular residues, mutant rNaD1(R40E) demonstrated an extremely reduced activity in the U937 cells. Thus, NaD1 leads the cancer cells to death through PIP_2_-mediated membrane binding, blebbing, focal permeabilization, and irreversible rupture [143,144].

A newly discovered defensin peptide obtained from the seeds of the white cloud bean (*Phaseolus vulgaris*) has been unearthed as a strong mitogenic agent after proper isolation and purification. This tiny peptide full of cysteine has approximately 7458 Daltons as its molecular mass. Its primary and most notable function is the facilitation of the apoptosis of the target cells. Experiments have been able to clearly demonstrate the growth-promoting effects on the two cell lines: it more than doubles MCF-7 human breast adenocarcinoma cells’ growth and division rate, and is an excessively potent mitogen for murine splenocytes, which implies its role in lymphocyte activation and expansion. Although the mechanism of its growth-promoting activity is not yet fully established, it is known to be separate from the cytotoxic action of many plant defensins and is thought to involve cell surface receptor-specific interaction, thereby leading to the initiation of pro-proliferative intracellular signaling pathways. The simultaneous action on both tumor and immune cell communicates its intricate biological role. Its strong effect on splenocytes, which are the main players in the adaptive immune response, is an indication of a significant function in immune modulation, probably boosting the body’s immunological surveillance and responsiveness. The white cloud bean defensin’s idiosyncratic combination of attributes makes it a very attractive area of research for new immunostimulatory therapeutics or as a biochemical tool for investigating cell cycle regulation [145]. Vulgarinin, an antifungal peptide with a molecular weight of about 7 kDa, was obtained from haricot bean seeds (Phaseolus vulgaris) and is a significant inhibitor for the proliferation of MCF-7, L1210, and M1 cell lines. This protein has two biological activities—antifungal and anti-proliferative. The plant’s potent defensins were thus discovered as possible therapeutic agents [146]. Cloud bean defensin, a 7.3 kDa peptide, is extracted from cloud bean (Phaseolus vulgaris) seeds as a defenisin-like antifungal peptide. The anticancer activity of this peptide is strong against the leukemia cell line L1210, where the needed concentration to kill 50% cells (IC_50_) is 10 μM. However, MBL2 lymphoma cells are also susceptible but show lower sensitivity, with an IC_50_ of 40 μM [147]. The same 7.1 kDa defensin-like peptide “Nepalese” was isolated from red beans (Phaseolus angularis) of Nepal. The peptide’s potency for the inhibition of growth in L1210 and MBL2 cancer cell lines corresponds to its antifungal activity. Its IC_50_ values are thus 15 μM and 60 μM, respectively [148]. Gymnin, a 6.5 kDa defensin-like peptide obtained from the Yunnan bean (Gymnocladus chinensis Baill), is a potent antifungal agent and possesses broad anti-proliferative properties. L1210, HepG2, and M1 cells are among those that are targeted by this activity. Furthermore, it inhibits the reverse transcriptase of human immunodeficiency virus-1, and the IC_50_ for this inhibition is 200 μM [149]. Table 2 outlines the anticancer activity of plant defensins.

The three cyclotides (varv A, varv F, and cycloviolacin O2) that come from *Viola arvensis* Murr. and *Viola odorata* L. showed strong cytotoxicity that was dependent on the dose across a range of ten human tumor cell lines. Cycloviolacin O2 was the strongest, having IC_50_ values of a very low sub-micromolar range (0.1–0.3 µM) [150]. The cycloviolacin O2 (CyO2) extracted from Viola odorata demonstrated remarkable anticancer effects through the staggering of specific cancer cell membranes. The compound was active against MCF-7 (with IC_50_ = 3.17 µM) and its drug-resistant subline MCF-7/ADR (with IC_50_ = 3.27 µM) at the same time. However, aliquots of CyO2 were much stronger than doxorubicin in terms of the sensitization of resistant cancer cells. The mechanism that disrupted the membrane was selective for tumor cells and did not affect normal human brain endothelial cells. New cyclotides from Psychotria leptothyrsa also enhanced doxorubicin toxicity, thus placing cyclotides among the potential drugs effective in the treatment of drug-resistant breast cancer. The findings of this research mark the first reported case of antitumor activity for cyclotides derived from the Rubiaceae family, namely the novel cyclotides psyle A, C, and E isolated from Psychotria leptothrysa var. longicarpa. All three cyclotides showed strong and dose-dependent cytotoxicity against the MCF-7 and MCF-7/ADR-resistant subline, but were always less potent against the resistant cells. In the MCF-7 line, psyle E displayed the highest potency (IC_50_ = 0.64 µM) and coincided with doxorubicin in potency (IC_50_ = 0.64 µM), followed by psyle C (IC_50_ = 2.98 µM) and psyle A (IC_50_ = 7.77 µM).

In the MCF-7/ADR subline, the same effectiveness was noted, with the determined IC_50_ values being 1.73 µM for psyle E, 8.7 µM for psyle C, and 12.0 µM for psyle A [151]. The cytotoxicity of Cycloviolacin O2 is critically determined by its surface charge and structural integrity. Chemical transformations indicated that one glutamic acid residue is very important since its methylation resulted in a 48-fold reduction in activity. Covering the single arginine did not change activity, while the acetylation of the two lysines led to a 3-fold decrease in activity. The simultaneous modification of the arginine and lysines resulted in a 7-fold decrease in activity. It is necessary for the disulfide-bonded core to be intact since the whole native structure is needed for its potent cytotoxic effect, as was shown by the inactivity of individual backbone loops [152]. Cycloviolacin O2 mainly disrupts the cell membrane rapidly to exert its potent cytotoxic effect. The experiment performed with human lymphoma and HeLa cells showed that the cell membrane of the affected cells is disintegrated within minutes of exposure. This was demonstrated through the concentration-dependent release of internal contents from both calcein-loaded cells and synthetic liposomes, which was an indication of the activity being concentration-dependent. The activity is particular, showing that cycloviolacin O2 directly targets the lipid membrane and gets through it.

The first thing that comes to mind is that the strong cytotoxic nature of cyclotides is largely attributed to the membrane-seizing effect as a primary mechanism. The selective toxicity towards cancer cells over normal ones in vitro is one of the most attractive aspects of cycloviolacin O2. Nevertheless, this potency did not appear in a mouse tumor model, which might be due to rapid clearance or ineffective distribution to the tumor site. On top of that, while mice were eventually killed at 2 mg/kg, none of the 1.5 mg/kg dose group displayed any discomfort. All these confounding in vivo results notwithstanding, their original mechanism and great stability still flirt with the possibility of being new antitumor agents [152,153,154]. Moreover, eight new cyclotides (Viphi A–H) extracted from Viola philippica have been revealed, increasing the already existing structural diversity of this stable peptide family. Several among them have been shown to be cytotoxic against MM96L, HeLa, and BGC-823 cancer cell lines, but their effects were usually highly sequence-dependent. To illustrate, Viphi D and Viphi E turned out to be totally inactive against the BGC-823 gastric cancer line, which highlights the subtle sequence variations that are very crucial for determining bioactivity. Such results are considered to give us very important structure–activity relationships for the purpose of drug design; at the same time, they help us to pinpoint active ingredients in traditional Chinese medicine [155].

The investigation of Mishra et al. [156] revealed a new cyclic octapeptide, cyclosaplin, which was extracted and isolated from somatic seedlings of sandalwood (*Santalum album* L.), having 858 Da as its molecular weight and a cyclic sequence of RLGDGCTR. This peptide was found to have an IC_50_ value of 2.06 μg/mL and, therefore, was confirmed to be a potent inhibitor of the proliferation of the MDA-MB-231 human breast cancer cell line. By using cyclosaplin as one of the agents, it was revealed through genesis studies that the compound restrains the cell division of cancer cells along with the processes of caspase-3 activation and the fragmentation of DNA, the loss of potential across mitochondria membrane, and finally cell death. The molecular modeling and docking studies corroborated the significant binding affinity of the peptide not just to EGFR but also to procaspase-3, while co-localization studies put forward that cyclosaplin sensitizes the cancer cells possibly through binding to EGFR and the activation of apoptotic pathways [156]. The cyclotide MCoTI-I, obtained from Momordica cochinchinensis, was investigated for its action against cancer cell lines such as LNCaP and HCT116 [81]. It was developed into a variant named MCo-PMI, which was designed to effectively bind and inhibit the Hdm2 and HdmX proteins, which are responsible for the deactivation of the p53 tumor suppressor pathway. This engineered cyclotide not only binds with both targets possessing low nanomolar affinity but also is very stable in serum, and thus it is capable of reactivating the p53 pathway, leading to the death of the wild-type p53 cancer cells both in vitro and in vivo.

The implantation of MCoTI-I cyclotide allows the development of drugs targeting intracellular protein–protein interactions in cancer [157]. The research that was performed on the herb *Hedyotis biflora*, a traditional Chinese medicinal herb, brought to light five new cytotoxic cyclotides. These cytotoxic cyclotides were named hedyotide B5 (HB5) to B9. Therefore, the known cyclotide family from this plant now includes the two previously identified HB1 and HB2, along with the others. The peptide sequences have been confirmed by Edman degradation and the gene-cloning approach. MTT assay results showed that all the hedyotides obtained had a strong cytotoxic effect on four pancreatic cancer cell lines. Out of those, HB7 had the highest potency. Commercial trials also suggested that HB7 held back cell migration and invasion in capan-2 cells. What is more, an in vivo xenograft experiment demonstrated that HB7-treated tumors were significantly smaller and lighter compared to control ones receiving no treatment [158]. Six novel cyclotides, vaby A–E and varv E, were among those that surfaced in a study aiming to determine the diversity of cyclotides, with their origin being the East African highlands violet, or the Viola abyssinica. Of these, vaby A and vaby D showed a promising degree of cytotoxicity against the U-937 lymphoma cell line. vaby A, B, and C have a commonality in that they have a unique structural attribute: the presence of an alanine amine in the second loop of their cyclic backbone. Isolation and characterization were achieved through HPLC, mass spectrometry, and quantitative amino acid analysis, affirming that the plant is a rich source of both structurally distinct and biologically active cyclotides [159]. Research involving the tropical medicinal plant Clitoria ternatea (Fabaceae) has resulted in the identification of 15 heat-stable cysteine-rich peptides (CRPs), out of which 12 have been recognized as novel sequences referred to as cliotides T1–T12. Extensive studies have ascertained that these cliotides are genuine cyclotides and not linear A1b peptides, judging by their sequence similarity, cyclic cystine knot structure, and disulfide connectivity. Thecliotides showed interaction with membranes and proved to be biologically active by killing *E. coli* and HeLa cancer cells. The cliotides 1–4 exert a remarkable anticancer effect on HeLa cells. Isolation of cyclotides in *Psychotria leptothyrsa* was performed and obtained six novel peptides labeled psyles A–F. Among these, psyle C is the first known linear cyclotide variant in the Rubiaceae family. The cytotoxicity of psyles A, C, and E was tested against the human U937-GTB lymphoma cell line, leading to IC_50_ readings of 26 µM, 3.50 µM, and 0.76 µM, respectively. Such findings not only increase the number of known rubiaceous cyclotides but also show that a linear structural variant can still be very cytotoxic [160,161]. The alpine violet Viola biflora has been revealed as a new cyclotide resource, presenting 11 novel sequences characterized as vibi A–K. For protein isolation, a combined approach of MS/MS sequencing and cDNA library screening using a degenerate primer for a conserved ER signal motif was adopted. Surprisingly, the methods exhibited a large difference, since only one isolated cyclotide (vibi D) was found to be directly matched to a cDNA clone. To determine bioactivity, the cytotoxic efficacy of specific vibis was determined by the cytotoxicity of vibis. The bracelet-type cyclotides vibi E, G, and H were found to be very cytotoxic to a lymphoma cell line, and their IC_50_ values were between 0.96 and 5.0 µM. In contrast, the Möbius cyclotide vibi D was found to be non-cytotoxic even at 30 µM, which is a high concentration, thus showing the connection between cytotoxicity and cyclotide subclass and structure very clearly [162]. The cytotoxicity and chemosensitizing capability of seven cyclotides (cliotides CT-2, CT-4, CT-7, CT-10, CT-12, and CT-19) from Clitoria ternatea were tested in paclitaxel-resistant lung cancer cells (A549/paclitaxel) and their parent line (A549). The selected cliotides were found to be significantly anticancerous and able to increase the effectiveness of paclitaxel, thus acting as very powerful chemosensitizers by dramatically lowering the IC_50_ of the chemotherapeutic agent when given in combination treatments. These bioactivities were, however, very closely associated with the net charge of the cyclotides, hence the hypothesis of electrostatic interactions being the means of their action [163]. A study by Burman et al. [164] established that the membrane-disrupting activities of cyclotides are greatly influenced by the lipid composition. Cycloviolacin O2 (cyO2) managed to thoroughly and selectively disrupt anionic membranes, while kalata B1 and kalata B2 obtained from Oldenlandia affinis were less lytic against all model membranes tested, even though they had previously been shown to possess cytotoxicity in cell lines like U-937 GTB, HT-29, and Ht116. The unnatural neutralization of the charged residues in cyO2 gave rise to surprising context-dependent effects: the Glu6 methyl ester derivative of cyO2 was found to be more potent on standard model membranes but suffered a 50-fold loss of activity in Escherichia coli lipid membranes. This particularity was ascribed to cyO2’s distinct feature of selectively drawing out certain phosphatidylethanolamine lipids (PE-C16:0/cyC17:0 and PE-C16:0/C18:1) from the membranes, which was not the case for kalata B1/B2. Table 3 illustrates in a straightforward manner the cancer suppressing mechanisms of both cyclic peptides and cyclotides.

## 6. Plant Anticancer Peptides as Functional Food Ingredients

The growing interest in plant anticancer peptides (PACPs) extends beyond their potential as pharmaceutical drugs. Many PACPs originate from common food plants and can be consumed as part of the diet, positioning them as promising candidates for functional foods and nutraceuticals. We discuss the integration of PACPs into food products, addressing definitions, natural occurrence, matrix interactions, technological considerations, regulatory aspects, and a clear distinction between pharmaceutical and food-relevant peptides. Furthermore, the increasing consumer demand for natural health-promoting ingredients has accelerated research into plant-derived bioactive peptides as food additives. From a commercial perspective, PACP-enriched functional foods represent a growing segment within the global nutraceutical market, offering opportunities for product differentiation and added value.

Functional foods are foods that provide health benefits beyond basic nutrition, while nutraceuticals are bioactive compounds (often isolated or purified) that can be used in dietary supplements or functional formulations. PACPs can be incorporated into both categories: they can be present naturally in whole foods (e.g., lunasin in soy [79]) or be enriched via processing (e.g., protein hydrolysates added to cereal bars). Depending on their potency, safety profile, and intended use, PACPs can be classified into two broad groups: Peptides with high potency (often nanomolar to low micromolar IC_50_) that typically require purification, parenteral administration, and rigorous clinical development. Their use is primarily oriented toward drug development for cancer therapy. Examples include cycloviolacin O_2_ [150,151,152,153,154], viscotoxins [128,129,130,131], NaD1 [143,144], and the cyclopeptide RA-V [99].

Peptides derived from edible sources that exhibit moderate anticancer activity are generally recognized as safe (GRAS) when consumed as part of food, and can be delivered orally via whole foods or hydrolysates. These are often obtained by the enzymatic hydrolysis of food-grade proteins and can be incorporated into everyday food products. Representative examples include lunasin [79], rice bran pentapeptide [84], mung bean peptides [88], corn peptides [102,120], and sunflower seed peptides [105]. Table 4 provides a clear distinction between these two categories. An important additional consideration is the dosage level: pharmaceutical peptides require precise, often high doses, whereas food-relevant peptides exert their effects gradually through sustained dietary intake. Moreover, the regulatory pathways for these two categories differ fundamentally, influencing the investment and timeline required for market entry. Table 4 provides a clear distinction between these two categories with representative examples.

### 6.1. Naturally Occurring PACPs in Common Foods

Many plant anticancer peptides are not present in an active form in raw plant tissues but are encrypted—that is, they are hidden within the amino acid sequences of larger precursor proteins such as globulins, albumins, and glutelins [79,80,81]. These encrypted peptides can be released through proteolytic hydrolysis during food processing (e.g., fermentation, enzymatic treatment) or during gastrointestinal digestion after consumption [91,92]. This characteristic makes staple foods and their by-products ideal sources for functional food development. For example, lunasin is naturally present in soybeans and survives mild cooking, making it bioavailable from tofu, miso, and soy beverages [79]. Similarly, rice bran, a major by-product of rice milling, contains the pentapeptide EQRPR, which is released upon enzymatic hydrolysis and shows broad-spectrum anticancer activity [84]. Legumes such as mung bean, common bean, and chickpea are rich in peptides that induce apoptosis or cell cycle arrest after simulated digestion [80,85,88]. Pseudocereals like quinoa and amaranth have gained attention because their proteins release peptides with anti-metastatic and antioxidant properties under gut-like conditions [91,92]. Oilseeds (sunflower, hemp, perilla) and nuts (walnut) also provide stable peptides that can be incorporated into bakery, snack, or confectionery products [83,97,98,105]. Table 5 summarizes key food-relevant PACPs, their plant sources, anticancer mechanisms, food matrix compatibility, and stability characteristics.

### 6.2. Food Matrix Interactions and Their Influence on Bioactivity

When PACPs are incorporated into food products, they interact with other components of the matrix, which can affect their stability, bioactivity, and bioavailability. Many plant-based foods contain polyphenols that can form non-covalent complexes with peptides. Such interactions may protect peptides from proteolytic degradation but may also mask their active sites, reducing activity. Conversely, synergistic effects have been reported where polyphenols and peptides together enhance antioxidant or anticancer actions (Cowpea peptides with polyphenols [107]). Peptides can bind to soluble or insoluble fibers, which may influence their release during digestion. Encapsulation within fiber matrices can provide controlled release and protection from gastric conditions. The presence of fats can affect the emulsification and micellization of hydrophobic peptides, potentially altering their absorption. Lipid-based delivery systems (e.g., liposomes) are being explored to improve the bioavailability of food-derived peptides. Competing proteins in the food matrix can affect peptide release during digestion. Processing methods that selectively hydrolyze specific protein fractions can enhance the yield of bioactive peptides [81,88]. In addition to these interactions, the food matrix can influence the peptide’s conformational stability; for example, certain polysaccharides may induce partial unfolding that either enhances or reduces bioactivity. Recent studies have also highlighted that the order of addition during food processing (e.g., mixing peptides before or after heat treatment) can dramatically alter matrix–peptide binding and final bioactivity.

### 6.3. Technological and Sensory Considerations for Food Applications

For successful commercialization as functional food ingredients, PACPs must meet several technological and sensory criteria. One of the most critical factors is heat stability, as peptides intended for incorporation into baked goods, extruded cereals, or pasteurized beverages must withstand thermal processing. Cyclotides and many defensins are exceptionally heat stable due to their disulfide-bonded structures [136,137,138,139,140,141,142,143,144,145,146,147,148,149,150,151,152,153,154], whereas linear peptides like lunasin [79] may require milder processing or post-processing addition. Corn peptides [102,120] and rice bran peptides [84] show good heat stability, making them suitable for baked goods and extruded cereals. Short-time high-temperature treatments, such as those used in snack food production, are generally less detrimental to peptide integrity than prolonged heating. For heat-sensitive peptides, encapsulation in heat-protective matrices (e.g., maltodextrin or modified starches) has emerged as a practical industrial solution.

pH stability is another key consideration, as food products range from acidic (beverages, yogurts, pH 3–5) to neutral (cereal bars, baked goods, pH 6–7). Peptides that remain active across a broad pH range are more versatile. Many legume-derived peptides [80,85,88] maintain activity at neutral to slightly acidic pH, while some, such as amaranth peptides [91], are active across a wider range. For acidic beverages (e.g., fruit juices or sports drinks), peptide aggregation due to low pH can be mitigated by adding stabilizers such as pectin or carboxymethylcellulose. Conversely, in neutral high-protein bars, the Maillard reaction between peptides and reducing sugars may occur during storage, potentially altering both taste and bioactivity.

Taste and odor pose significant challenges, as hydrophobic peptides can impart bitterness—a common issue in protein hydrolysates. Several debittering strategies exist, including the selective removal of hydrophobic peptides using adsorption resins, encapsulation with cyclodextrins or liposomes, masking with flavors (e.g., fruit flavors for beverages, cocoa for bars), and enzymatic debittering using exopeptidases. Recent advances in fermentation-assisted hydrolysis have shown that certain lactic acid bacteria can selectively remove bitter peptides while preserving anticancer activity. Furthermore, the use of allulose or other rare sugars has been reported to effectively mask bitterness without adding calories, making them attractive for functional beverage applications.

Texture modification is another important consideration, as the addition of peptide fractions can affect viscosity, gelation, and mouthfeel. Formulation adjustments, such as the addition of hydrocolloids or emulsifiers, may be required to maintain acceptable sensory properties. Peptide hydrolysates with high-molecular-weight distributions tend to increase viscosity more than low-molecular-weight fractions, which can be exploited to improve texture in yogurts or puddings. In contrast, for clear beverages, ultrafiltration is often used to remove larger peptide aggregates that cause turbidity, ensuring a visually appealing product.

Finally, processing compatibility is essential for industrial adoption. Industrial food processing operations such as high-pressure processing, homogenization, and spray drying are generally compatible with peptide hydrolysates. The extraction methods discussed in Section 3 (UAE [60,61,62,63,64], MAE [57,58,59], HHP [68,69,70], PEF [65,66,67]) are already used in the food industry, making the production of peptide-rich hydrolysates commercially feasible. Among these, spray drying is particularly attractive because it yields a stable powder that can be easily incorporated into dry mixes, protein bars, or ready-to-drink formulations. However, care must be taken to avoid nozzle clogging when hydrolysates contain high amounts of hydrophobic peptides; the use of carrier agents such as gum Arabic or lecithin can mitigate this issue.

### 6.4. Bioavailability and Digestion in the Food Context

Unlike pharmaceutical applications where peptides are often administered intravenously, the oral consumption of functional foods relies on gastrointestinal digestion and absorption. Several studies have employed in vitro simulated gastrointestinal digestion (e.g., for amaranth [91], quinoa [92], sunflower [105]) to demonstrate that anticancer peptides are released from food proteins under conditions mimicking the human gut. This approach is critical for substantiating functional food claims. Many PACPs are inactive within the intact parent protein and become bioactive only after enzymatic hydrolysis (e.g., by pepsin, trypsin, chymotrypsin) [80,81,88,91]. This aligns perfectly with the functional food paradigm, where the food matrix serves as a natural precursor that yields active peptides upon consumption. Factors affecting bioaccessibility include food matrix complexity, peptide length, and the presence of enzyme inhibitors. Small peptides (di- and tripeptides) can be absorbed via PepT1 transporters, while larger peptides may undergo paracellular transport or transcytosis. Some cyclic peptides (cyclotides) are remarkably resistant to proteolysis but show low oral absorption, limiting their food applicability [150,151,152,153,154]. For food-relevant PACPs, studies should include Caco-2 cell monolayers or animal models to assess intestinal permeability and bioavailability. An emerging concept is the role of the gut microbiota in further metabolizing peptide fragments, potentially generating new bioactive species that were not present in the original hydrolysate. Moreover, the timing of peptide intake relative to meals (i.e., fed vs. fasted state) has been shown to significantly influence bioaccessibility, a factor that should be considered when designing realistic consumption protocols for functional foods.

## 7. Bioavailability and Stability of Plant Anticancer Peptides

The clinical and functional food translation of PACPs is critically hindered by challenges related to their bioavailability and stability. While PACPs exhibit potent anticancer activity in vitro, their efficacy in vivo particularly after oral administration is often limited by rapid degradation, poor absorption, and extensive first pass metabolism [164,165,166].

### 7.1. Intrinsic Stability and Structural Determinants

The susceptibility of PACPs to proteolytic degradation is largely dictated by their primary and three-dimensional structures. Linear peptides are generally rapidly degraded by proteases present in the gastrointestinal tract (pepsin, trypsin, chymotrypsin) and bloodstream (serine proteases, carboxypeptidases). In contrast, peptides with constrained structures exhibit enhanced stability [167,168]. Cyclotides possess a cyclic cystine knot (CCK) topology that confers exceptional resistance to thermal, chemical, and enzymatic degradation. This makes them one of the most stable peptide families known, with the ability to survive simulated gastrointestinal digestion and even prolonged exposure to serum proteases [169]. Defensins and thionins, stabilized by multiple disulfide bonds, also demonstrate considerable resistance to proteolysis, although their linear termini may still be vulnerable to exopeptidases [170]. Linear α helical and random coil peptides are typically the least stable, requiring modification or formulation strategies to achieve sufficient in vivo half-life [171].

### 7.2. Gastrointestinal Digestion and the Encrypted Peptide Paradigm

For orally administered PACPs, whether as purified compounds or as components of functional foods, the gastrointestinal (GI) tract represents the first major barrier. The harsh acidic environment of the stomach (pH 1.5–3.5) can denature peptides, while pepsin initiates proteolytic cleavage. Subsequent exposure to pancreatic enzymes (trypsin, chymotrypsin, elastase, carboxypeptidases) in the small intestine further degrades peptides into smaller fragments or free amino acids [172]. Importantly, this digestive process is not uniformly detrimental. Many PACPs are encrypted within larger precursor proteins and are only released as active peptides upon enzymatic hydrolysis during GI digestion or food processing. Studies on amaranth, quinoa, and sunflower seeds have demonstrated that in vitro simulated GI digestion releases bioactive peptides with anticancer activity from intact food proteins. This paradigm is central to the functional food concept, where the food matrix serves as a natural delivery system that yields active peptides upon consumption [173].

### 7.3. Absorption Mechanisms and Systemic Bioavailability

Even if a peptide survives the GI tract, its absorption into the bloodstream is limited by several factors such as molecular size, hydrophilicity and charge, as well as first-pass metabolism. Peptides larger than 3–5 kDa are poorly absorbed across the intestinal epithelium, with transport occurring primarily via paracellular diffusion (for small peptides) or active transcellular transport (e.g., via PepT1 for di and tripeptides). Most PACPs are in the 1–10 kDa range, placing them at the threshold of efficient absorption [174,175]. The cationic nature of many PACPs can facilitate interaction with negatively charged cell membranes but does not guarantee transcellular transport. Hydrophilic peptides often lack the lipophilicity required to cross the lipid bilayer. Peptides that are absorbed via the portal vein are subject to extensive metabolism in the liver, where hepatic proteases and cytochrome P450 enzymes may further degrade or modify them before they reach systemic circulation [176,177].

### 7.4. Strategies to Enhance Stability and Bioavailability

A range of innovative strategies have been developed to overcome the bioavailability limitations of PACPs. These approaches can be broadly categorized into chemical modification, formulation technologies, and delivery systems.

#### 7.4.1. Chemical Modification and Peptide Engineering

Cyclization, such as head-to-tail cyclization, eliminates the N and C termini that are the primary targets of exopeptidases while often increasing conformational rigidity, a strategy exemplified by naturally occurring cyclotides and successfully applied to engineer stable analogs of linear peptides [178,179]. Introducing non-natural amino acids for instance, substituting L-amino acids with D-amino acids, β-amino acids, or other peptidomimetics, confers resistance to stereospecific proteases, dramatically increasing plasma half-life while preserving or even enhancing bioactivity [180,181]. PEGylation, the covalent attachment of polyethylene glycol chains, increases the hydrodynamic radius, thereby reducing renal clearance and protecting against proteolytic degradation, with PEGylated peptide analogs showing improved pharmacokinetics in preclinical models [182]. Finally, N- and C-terminal capping, such as acetylation of the N-terminus and amidation of the C-terminus, shields the peptide from exopeptidase activity, offering a simple yet effective strategy for enhancing stability [183].

#### 7.4.2. Nanotechnology Based Delivery Systems

Nanocarriers protect anticancer peptides from degradation, enable controlled release, and allow tumor targeting. Phospholipid bilayers encapsulate hydrophilic or hydrophobic peptides, enhancing cellular uptake and enzymatic protection; liposomal formulations have shown improved efficacy and reduced toxicity. Biodegradable polymers like PLGA, chitosan, and alginate protect against GI degradation and enable sustained release. Surface functionalization with ligands such as folic acid enables active targeting. Lipid-based systems offer stability and controlled release using GRAS excipients. Inorganic carriers like mesoporous silica, gold nanoparticles, and carbon nanotubes provide high loading capacity and stimuli-responsive release (e.g., pH or enzymatic triggers) [184,185,186].

#### 7.4.3. Encapsulation and Food Matrix Engineering for Functional Foods

Spray drying and freeze drying, along with microencapsulation using maltodextrin or gum Arabic, produce peptide-loaded powders with improved storage stability. Emulsion systems such as water-in-oil or double emulsions encapsulate hydrophilic peptides, protecting them from gastric conditions and enabling intestinal release. Hydrogels from alginate, pectin, or proteins provide gastric protection and small intestine release. Co-encapsulation with protease inhibitors like soybean trypsin inhibitor or chitosan reduces GI degradation, a strategy well suited for functional food formulations [187,188].

#### 7.4.4. Alternative Administration Routes

Alternative routes bypass the GI barrier: intravenous injection provides 100% bioavailability but requires medical supervision; subcutaneous or intramuscular injections enable sustained release over days to weeks. Transdermal methods (iontophoresis, microneedles, chemical enhancers) offer non-invasive absorption. Pulmonary delivery via inhalable nanoparticles or dry powders targets the lungs, relevant for lung cancer therapy. Nasal and buccal routes access highly vascularized mucosa, avoiding GI degradation and first-pass metabolism [189,190].

### 7.5. Local vs. Systemic Effects: Implications for Cancer Therapy

An important consideration in bioavailability discussions is whether systemic absorption is necessary for therapeutic efficacy. Some PACPs may exert local effects in the GI tract without requiring systemic exposure. Peptides that remain intact in the colon (e.g., some cyclotides, defensins, or encapsulated formulations) can directly target colorectal tumors without the need for absorption. Peptides that interact with immune cells in the intestinal mucosa may elicit systemic immune responses even if they do not enter the circulation intact. Thus, strategies to enhance bioavailability should be tailored to the target cancer type and the intended mechanism of action. For systemic cancers (e.g., breast, lung, liver), achieving adequate plasma concentrations is essential; for GI cancers, local delivery may suffice [173,174,175,176].

### 7.6. Preclinical Models for Bioavailability Assessment

The reliable prediction of human bioavailability requires appropriate preclinical models. Standardized protocols (e.g., INFOGEST) provide valuable data on peptide stability and release from food matrices. These models are widely used in food science to assess the potential of protein hydrolysates as functional ingredients. Ex vivo models, using chambers with intestinal tissue explants, can measure the transepithelial transport of intact peptides. Rodent models remain the gold standard for assessing pharmacokinetics (absorption, distribution, metabolism, excretion) and oral bioavailability. However, species differences in GI physiology and protease expression must be considered when extrapolating to humans [191,192,193].

## 8. Challenges and Limitations in the Development of Plant-Derived Anticancer Peptides

Despite the immense potential of PACPs as therapeutic agents and functional food ingredients, their translation from laboratory research to real-world applications faces several significant challenges. Acknowledging these limitations is essential for guiding future research efforts and setting realistic expectations for industrial and clinical development.

### 8.1. Production Scalability and Cost

The efficient and cost-effective production of PACPs remains a major hurdle. Traditional extraction from plant biomass suffers from low yields, batch-to-batch variability, and dependence on seasonal and geographical factors. While chemical synthesis (solid phase peptide synthesis, SPPS) is feasible for short peptides (<30–40 amino acids), it becomes prohibitively expensive for longer sequences or large-scale manufacturing. Recombinant expression in microbial hosts (*Escherichia coli*, *Pichia pastoris*) offers a scalable alternative, but challenges such as the toxicity of the expressed peptide to the host, improper folding, and low yields persist, especially for disulfide-rich peptides like cyclotides and defensins. Cell-free protein synthesis is an emerging technology that may circumvent some of these issues, but it is still in early development and not yet cost competitive for bulk production [194,195,196,197].

### 8.2. Technological Costs and Processing Constraints

Beyond production, downstream processing—including purification, formulation, and quality control—adds substantial costs. High-performance liquid chromatography (HPLC) is often required to obtain pure peptides, but it is not easily scalable. Membrane filtration, precipitation, and other unit operations can be used for crude fractions, but achieving the purity required for pharmaceutical applications drives up expenses. For functional food applications, cost constraints are even stricter, as margins are lower than for therapeutics. Additionally, novel processing technologies (e.g., ultrasound assisted extraction, pulsed electric fields) require capital investment and optimization to be implemented at industrial scales [198,199,200]

### 8.3. Raw Material Standardization and Supply Chain Variability

As the natural source of PACPs, plant biomass is inherently variable. Factors such as cultivar, growing conditions, harvest time, and post-harvest processing can significantly affect the yield and composition of bioactive peptides. This variability poses a challenge for both research reproducibility and commercial manufacturing. Establishing standardized cultivation protocols, defining chemotypes, and using well-characterized reference materials are necessary to ensure consistent quality. For peptides obtained as by products (e.g., from soybean meal, rice bran, corn gluten meal), the availability and quality of the starting material can fluctuate depending on the primary food processing industry, requiring robust quality management systems [201,202].

### 8.4. Safety and Toxicological Considerations

Despite the selective cytotoxicity of many plant anticancer peptides (PACPs) toward cancer cells, safety concerns remain significant. Peptides like thionins and defensins can cause hemolysis or damage normal cells; pyrularia thionin and Thi2.1, for example, are toxic to non-cancerous cells, and even selective peptides may accumulate in healthy tissues or trigger immune reactions. Key issues include immunogenicity (non-human peptides may elicit neutralizing antibodies or allergies, especially with repeated dosing), genotoxicity and chronic toxicity (long term safety data are lacking, and regulatory toxicology studies are resource intensive), and hemolytic activity (many cationic membrane active peptides lyse red blood cells). For functional food applications, safety thresholds are even stricter due to daily, widespread consumption; the effects of chronic low-dose exposure remain largely unknown, requiring rigorous post-market surveillance [203,204,205,206,207,208,209].

### 8.5. Regulatory Hurdles: FDA, EFSA, and Other Frameworks

The regulatory landscape for PACPs varies by use (drugs, supplements, functional foods) and jurisdiction. For pharmaceuticals, the FDA (US) requires an IND application, preclinical studies, and Phase I–III trials (10–15 years, high cost), while the EMA oversees similar centralized approval in the EU. Dietary supplements in the US fall under DSHEA; NDIs may be needed for ingredients not marketed before 1994. In the EU, supplements follow the Food Supplements Directive (2002/46/EC), with cancer prevention claims requiring strong evidence. Functional foods are regulated under general food law (e.g., FDA’s FD&C Act; EU’s Regulation 178/2002). Health claims like “reduces cancer risk” are strictly controlled: only authorized claims in the EU Register or FDA approved constitute qualified claims. A major barrier is the lack of standardized methods for characterizing PACPs in complex matrices, complicating submissions and quality control; harmonized analytical methods and validated alternatives to animal testing remain ongoing needs [210,211,212].

### 8.6. Intellectual Property and Commercial Viability

The patent landscape for natural derived peptides can be complex. While isolated and purified peptides with novel sequences can be patented, the presence of such peptides in traditional foods may limit the scope of intellectual property protection. Companies must navigate freedom to operate analyses and consider the cost of defending patents. Additionally, the relatively low profit margins for food products compared to drugs may deter investment in the extensive research required to bring a peptide-based functional food to market with substantiated health claims [213].

## 9. Future Perspectives

This review’s strong evidence brings to light the great potential of peptides from plants as a new-generation weapon of choice against cancer. Their differing structures, the targeting of cancer characteristics, and low toxicity levels make them suitable candidates to overcome the drawbacks of traditional chemotherapy drugs. Nevertheless, moving from laboratory to clinical use is a long and difficult process. Interdisciplinary innovation will be the key factor in the future of PACP development where the studies dealing with these difficulties will take place.

### 9.1. Overcoming Current Challenges

The major hurdles hindering the clinical translation of PACPs are threefold. The first are proteolytic stability and plasma half-life: linear peptides are rapidly degraded by proteases in the gastrointestinal tract and bloodstream, resulting in very low bioavailability. The second is oral bioavailability: due to their large molecular size and often hydrophilic nature, oral absorption is extremely poor, leaving parenteral administration as the only practical route. The third is scalable production: extracting and purifying peptides from plant biomass is low-yielding, costly, and environmentally demanding, limiting the large-scale supply required for clinical trials and market entry. Overcoming these challenges will require innovative formulation strategies, such as encapsulation and chemical modification, as well as the development of sustainable recombinant production systems. Only by addressing these three barriers can PACPs transition from promising laboratory candidates to clinically viable therapeutics.

### 9.2. Key Strategic Directions for Future Research

To overcome these challenges, future research must focus on key strategic areas. Through rational design, using walnut peptides PISLKSE, VSLP, and SHTLP as case studies, computational biology and molecular docking can create analogs with higher binding affinity to targets such as CASP3 and MMP9, along with improved pharmacological properties. Stability engineering involves incorporating non-natural D-amino acids, peptidomimetics, or N- and C-terminal modifications (e.g., acetylation, amidation) to render peptides resistant to exoproteases. Cyclization and grafting take advantage of the natural stability of cyclotides; their cyclic cystine knot (CCK) scaffold serves as a stable framework onto which biotherapeutic epitopes from linear peptides can be grafted, forming new chimeric molecules with dual functions.

#### 9.2.1. Advanced Delivery Systems

Nanotechnology-based delivery is arguably the most significant and promising path. Nanoparticles such as liposomes, polymeric nanoparticles, and micelles can act as carriers for PACPs, protecting them from degradation, enhancing tumor specificity via the enhanced permeability and retention (EPR) effect, and enabling controlled drug release. Conventional nanoparticles can be further functionalized with specific targeting ligands (e.g., antibodies, folic acid) that bind to receptors overexpressed on cancer cells (such as HER2 or the folate receptor), allowing active targeting and minimizing off-target effects. 

#### 9.2.2. Sustainable and Scalable Production

The sustainable and scalable production of PACPs can be achieved through several approaches. Recombinant expression systems move beyond plant extraction by developing efficient microbial factories (e.g., E. coli, Pichia pastoris) or plant cell cultures, enabling the consistent, high-yield, and scalable production of defined peptide sequences. Cell-free protein synthesis offers a rapid and flexible platform for producing peptides, including those with toxic or unstable characteristics, without the constraints of cellular viability. For shorter peptides, advances in automated solid-phase peptide synthesis (SPPS) provide a reliable chemical route for producing clinical-grade material.

#### 9.2.3. Expanding Mechanistic and Clinical Understanding

Future research must focus on elucidating precise mechanisms; while many mechanisms such as apoptosis and cell cycle arrest are proposed, deeper studies are needed to answer questions such as how peptides like LLPSY enhance cellular adhesion and what specific intracellular receptors exist for peptides that do not act via membrane disruption. Research should also focus on immuno-oncology by capitalizing on the immunomodulatory properties of peptides such as AGP and ABP from Abrus lectins, exploring their potential as cancer vaccine adjuvants or in combination with immune checkpoint inhibitors to reactivate the tumor microenvironment. Combination therapies should systematically evaluate PACPs as chemosensitizers, as seen with cycloviolacin O2 and cliotides; combining a low dose of a PACP with established chemotherapeutics could re-sensitize resistant tumors, reduce chemotherapy-related side effects, and improve overall efficacy. Finally, robust preclinical and clinical trials must prioritize the most promising candidates (e.g., lunasin, cycloviolacin O2, NaD1) for rigorous in vivo studies in relevant animal models, focusing not only on efficacy but also on comprehensive toxicology and pharmacokinetics, as these data are essential for advancing into human clinical trials.

## 10. Conclusions

This review demonstrates that small, cationic, amphipathic plant peptides—stabilized by α-helices, β-sheets, random coils, and cyclic disulfide-bonded frameworks—exhibit diverse anticancer mechanisms. These include apoptosis (intrinsic/extrinsic pathways), cell cycle arrest (G0/G1, S, G2/M), angiogenesis inhibition via MMP modulation, immune cell recruitment, interference with signaling pathways (PI3K/AKT, TGF-β), and the activation of Keap1/Nrf2 antioxidant defenses. Over 30 peptides from legumes (soybean lunasin, L/I-VPK; chickpea CPe-III-S), cereals (rice bran, corn), nuts (walnut PISLKSE, VSLP), and specialty plants (cycloviolacin O2, viscotoxins) show efficacy against breast, colon, liver, and leukemia cancers in vitro and in animal models. A key distinction emerges: potent targeted therapeutics (e.g., cycloviolacin O2, viscotoxins, RA-V) versus food-relevant peptides (e.g., lunasin, rice, corn, mung bean, sunflower peptides) with favorable safety profiles and activity in dietary forms, suitable for chemoprevention via functional foods. Major challenges remain—proteolytic instability, poor oral absorption, and scalable production—requiring the bioengineering of stable analogs, nanotechnology-based delivery, recombinant expression systems, and for food applications, improved processing stability, sensory optimization, and human intervention studies. Addressing these hurdles will translate PACPs into clinical therapeutics and commercial functional foods.

## Figures and Tables

**Table 1:** Anticancer activity of plant thionins.

Thionin Name	Source Plant	Cancer Cell Line/Model Tested	In Vitro/ In Vivo	Potency (IC_50_ or % Inhibition)	Key Findings & Proposed Mechanism	Selectivity (Cancer vs. Normal Cells)	Reference
Pyrularia Thionin	*Pyrularia pubera*	HeLa (cervical), B16 (mouse melanoma)	in vitro	50 μg/mL	Causes membrane disruption, depolarization, Ca^2+^ influx, and phospholipase A2 activation, leading to cell lysis.	Not selective; causes hemolysis (red blood cell lysis).	[127]
Viscotoxin B2	*Viscum coloratum*	Rat osteoblast-like sarcoma	in vitro	1.6 mg/L	Exhibits distinct cytotoxic activity; belongs to a subfamily known for specific cytotoxicity against tumor cells.	Suggested to be more specific for cancer cells (inferred).	[128]
Viscotoxin A3 (VA3)	*Viscum album* (inferred)	Not specified (mechanism studied)	Not specified	Not specified	Selectively targets phosphatidylserine (PS) lipids exposed on cancer cell membranes, causing membrane disruption and cell death.	Selective; 7–8× higher PS exposure on cancer cells.	[130,131]
Ligatoxin B	*Phoradendron liga*	ACHN (multidrug-resistant renal adenocarcinoma), U-937-GTB (lymphoma)	in vivo	3.2 μM (ACHN), 1.8 μM (U-937)	Potent against multidrug-resistant lines. Proposed novel mechanism: DNA-binding via a helix-turn-helix motif.	Not specified	[132]
Phoratoxin C	*Phoradendron tomentosum*	Panel of solid and hematological tumor cell lines; Primary patient-derived breast cancer cells	in vivo	0.16 μM	Most potent among phoratoxins. Shows differential activity.	Selective; 18× more effective against solid breast tumors than hematological cancers.	[133,134]
Phoratoxin F	*Phoradendron tomentosum*	Panel of solid and hematological tumor cell lines	in vivo	0.40 μM	Significant anticancer activity, but less potent than Phoratoxin C.	Differential activity (inferred from comparison to Phoratoxin C).	[133,134]
Thi2.1	*Arabidopsis thaliana*	MCF-7 (breast), A549 (lung), HeLa (cervical)	in vitro	94%, 29%, 38% inhibition	Conditioned media containing the thionin shows strong inhibitory effects on viability.	Not selective; highly cytotoxic to normal bovine mammary and endothelial cells.	[135]

**Table 2:** Anticancer activity of plant defensins.

Defensin Name	Source Plant	Mol. Mass (kDa)	Target Cancer Cell Line(s)	In Vitro/ In Vivo	Potency (IC_50_)	Mechanism/ Key Effects	Reference
Sesquin	*Vigna* *sesquipedalis* (Asparagus bean)	Not specified	MCF-7 (breast), M1 (leukemia)	in vitro	2.5 mg/mL	Inhibits proliferation. First reported plant defensin with anticancer activity.	[136]
Lunatusin	*Phaseolus lunatus* (Lima bean)	Not specified	MCF-7 (breast)	in vitro	5.71 µM	Inhibits proliferation. Non-selective: also inhibits cell-free translation, indicating toxicity to normal cells.	[137]
Limenin	Shelf bean	6.5	M1 (myeloma), L1210 (leukemia)	in vitro	Not specified	Inhibits DNA synthesis and reduces thymidine incorporation, suppressing proliferation.	[138]
Purple Pole Bean Defensin	*P. vulgaris* (Purple pole bean)	5.443	HepG2 MCF-7 HT29 SiHa (cervical)	in vitro	4.1 ± 0.8 µM (HepG2)	Selective toxicity: Potently inhibits cancer cells but spares normal WRL68 cells.	[139]
Coccinin	*P. coccineus* (Scarlet runner bean)	7	HL60, L1210 (leukemia)	in vitro	30–40 µM	Selective toxicity: Inhibits leukemia cells but spares normal mouse splenocytes.	[140]
Phaseococcin	*P. coccineus*	5.422	HL60, L1210 (leukemia)	in vitro	30–40 µM	Selective toxicity: Inhibits leukemia cells with no effect on normal splenocytes or protein synthesis.	[141]
Capsicum γ-Thionin	*Capsicum chinense* (Pepper)	Not specified	HeLa (cervical)	in vitro	100% inhibition (Conditioned media)	Selective toxicity: Completely inhibits HeLa viability but spares bovine endothelial cells. Effect confirmed with synthetic version.	[142]
NaD1	Nicotiana alata (Ornamental tobacco)	Not specified	HeLa U937 (lymphoma), PC3	in vitro	Not specified	Lytic mechanism: Binds PIP_2_ on plasma membrane, causes rapid blebbing, focal permeabilization, and complete cell lysis (LDH release).	[143,144]
White Cloud Bean Defensin	*P. vulgaris* (White cloud bean)	7.458	MCF-7 [Note: Acts as a mitogen, not cytotoxic]	in vitro	Not specified	Stimulates proliferation, acting as a potent mitogen. Believed to interact with cell surface receptors to trigger pro-proliferative signaling.	[145]
Vulgarinin	*P. vulgaris* (Haricot bean)	~7	MCF-7 L1210, M1 (myeloma)	in vitro	Not specified	Inhibits proliferation. Has dual antifungal and anti-proliferative activities.	[146]
Cloud Bean Defensin	*P. vulgaris* (Cloud bean)	7.3	L1210 MBL2 (lymphoma)	in vitro	10 µM (L1210), 40 µM (MBL2)	Antifungal peptide with anti-proliferative activity.	[147]
Nepalese	*P. angularis* (Nepalese red bean)	7.1	L1210 (leukemia), MBL2 (lymphoma)	in vitro	15 µM (L1210), 60 µM (MBL2)	Defensin-like peptide with antifungal and anti-proliferative activity.	[148]
Gymnin	*Gymnocladus chinensis* (Yunnan bean)	6.5	L1210 HepG2 M1	in vitro	Not specified	Antifungal and anti-proliferative activity. Also inhibits HIV-1 reverse transcriptase	[149]

**Table 3:** Cyclotides and cyclic peptides with anticancer properties.

Name (Type)	Size	Target Cell Lines/Effects	In Vitro/ In Vivo	Mechanism/Key Characteristics
Varv A, Varv F, Cycloviolacin O2 (Cyclotide)	Not specified	Panel of 10 human tumor cell lines	in vitro	Potent, dose-dependent cytotoxicity. CyO2 is most potent (IC_50_: 0.1–0.3 µM).
Cycloviolacin O2 (CyO2) (Cyclotide)	Not specified	MCF-7, MCF-7/ADR	in vitro	Membrane disruption & Chemosensitization. Permeabilizes cancer cell membranes. Enhances doxorubicin efficacy and internalization in resistant cells. Selective for tumor cells.
Psyle A, C, E (Cyclotide)	Not specified	MCF-7, MCF-7/ADR	in vitro	Dose-dependent cytotoxicity. Psyle E is most potent (IC_50_ = 0.64 µM in MCF-7). First antitumor cyclotides from Rubiaceae family.
Viphi A-H (Cyclotide)	Not specified	MM96L, HeLa, BGC-823	in vitro	Cytotoxicity is highly sequence-dependent (e.g., Viphi D & E inactive on BGC-823). Demonstrates how subtle changes affect bioactivity.
Cyclosaplin (Cyclic Octapeptide)	858 Da	MDA-MB-231	in vitro	Induces apoptosis. Causes mitochondrial membrane potential loss, DNA fragmentation, cell cycle arrest, and caspase-3 activation. Binds strongly to EGFR and procaspase-3.
MCoTI-I/MCo-PMI (Engineered Cyclotide)	Not specified	LNCaP, HCT116 (p53 wild-type)	in vitro	Inhibits intracellular PPIs. Engineered variant MCo-PMI targets Hdm2/HdmX proteins, reactivates p53 pathway, inducing apoptosis. High serum stability.
Hedyotide B5-B9 (HB7) (Cyclotide)	Not specified	Pancreatic cancer lines (Capan-2)	in vitro	Cytotoxicity & inhibits metastasis. HB7 is most potent, inhibits cell migration and invasion. Reduces tumor size/weight in vivo.
Vaby A, Vaby D (Cyclotide)	Not specified	U-937 lymphoma	in vitro	Significant cytotoxic activity. Vaby A, B, and C have a unique alanine residue in loop 2.
Cliotides T1-T12 (e.g., CT-1 to 4) (Cyclotide)	Not specified	HeLa, E. coli	in vitro	Membrane-active. Exhibit both antimicrobial and cytotoxic activity. Confirmed cyclic cystine knot (CCK) structure.
Psyles A–F (Psyle C is linear) (Cyclotide)	Not specified	U-937-GTB lymphoma	in vitro	Psyle C is first linear cyclotide in Rubiaceae but retains potency (IC_50_ = 3.50 µM). Psyle E is most potent (IC_50_ = 0.76 µM).
Vibi E, G, H (Bracelet) (Cyclotide)	Not specified	Lymphoma cell line	in vitro	Potent cytotoxicity (IC_50_: 0.96 to 5.0 µM). Activity is linked to cyclotide subfamily (Bracelet-type).
Vibi D (Möbius) (Cyclotide)	Not specified	Lymphoma cell line	in vitro	No cytotoxicity (even at 30 µM). Contrast with bracelet-type shows structure-activity relationship.
Cliotides (e.g., CT-2,4,7,10,12,19) (Cyclotide)	Not specified	A549, A549/paclitaxel	in vitro	Chemosensitization. Exhibit anticancer activity and enhance paclitaxel efficacy in resistant cells. Activity correlates with net charge.
Cycloviolacin O2 (cyO2) (Cyclotide)	Not specified	Model lipid membranes	in vitro	Lipid-specific membrane disruption. Potent and selective disruption of anionic membranes. Extracts specific phosphatidylethanolamine lipids, a unique mechanism.
Kalata B1, B2 (Cyclotide)	Not specified	U-937 GTB, HT-29, Ht116	in vitro	Less lytic on model membranes than cyO2, yet cytotoxic to cells. Suggests a possible secondary mechanism beyond lysis.

**Table 4:** Distinction between pharmaceutical and functional food/nutraceutical plant anticancer peptides.

Feature	Pharmaceutical Candidates	Functional Food/Nutraceutical Candidates
Primary source	Often from non-food plants (e.g., *Viola* [150,151,152,153,154], *Phoradendron* [133,134], *Nicotiana* [143,144])	Edible food plants (soy [79], rice [84], corn [102,120], legumes [80,85,88], quinoa [92])
Typical IC_50_	Low nanomolar to low micromolar (e.g., 0.1–3 µM [150])	Moderate (e.g., 0.15–20 mg/mL or 0.2–50 µM [84,88,113])
Required purity	High (purified single peptide)	Low to moderate (hydrolysates or peptide fractions acceptable)
Administration route	Parenteral (injectable) or topical	Oral (via food or supplement)
Regulatory pathway	FDA NDA/IND (drug approval)	GRAS notification, health claim substantiation
Stability requirement	In vivo stability (serum half-life)	Processing stability (heat, pH, storage)
Examples	Cycloviolacin O2 [150,151,152,153,154], Viscotoxins [128,129,130,131], NaD1 [143,144], RA-V [99], Phoratoxin C [133,134]	Lunasin [79], Rice bran pentapeptide [84], Mung bean peptides [88], Corn peptides [102,120], Sunflower peptides [105], Soybean L/I-VPK [113]

**Table 5:** Food-relevant plant anticancer peptides for functional food/nutraceutical development.

Peptide/Source	Plant Source	Key Anticancer Mechanism	Food Matrix Compatibility	Stability	Reference
Lunasin	Soybean (*Glycine max*)	Epigenetic (histone acetylation); apoptosis [79]	Soy products, beverages, tofu, miso	Moderate (heat-labile; survives some cooking)	[79]
Rice bran pentapeptide (EQRPR)	Rice bran (*Oryza sativa*)	Cell cycle arrest (broad-spectrum) [84]	Cereal bars, supplements, rice-based beverages	High	[84]
Mung bean peptides (VEG, PQG, LAF, EGA)	Mung bean (*Vigna radiata*)	Apoptosis; cell cycle arrest (S and G0/G1) [88]	Sprouts, hydrolysates, Asian noodle dishes	Moderate	[88]
Corn peptides (CPs)	Corn (*Zea mays*)	Apoptosis (mitochondrial); immunomodulation [102,120]	Corn-based foods, tortillas, snacks, cereals	High	[102,120]
Chickpea CPe-III-S (RQSHFANAQP)	Chickpea (*Cicer arietinum*)	p53 activation [85]	Hummus, flour, hydrolysates, ready meals	Not specified	[85]
Soybean L/I-VPK	Black soybean (*Glycine max*)	Caspase-3 binding; apoptosis [113]	Fermented soybean products, supplements	High (cyclic-like stability)	[113]
Sunflower peptides (D-8-K, T-11-E, P-12-V)	Sunflower (*Helianthus annuus*)	Keap1/Nrf2 activation; anti-inflammatory [105]	Snack foods, seed-based products, bakery	High	[105]
Quinoa peptides (IFQEYI, DVYSPEAG, etc.)	Quinoa (*Chenopodium quinoa*)	Colon cancer cell inhibition [92]	Gluten-free products, salads, breakfast cereals	Moderate	[92]
Amaranth peptides	Amaranth (*Amaranthus caudatus*)	Apoptosis; anti-metastasis; antioxidant [91]	Andean traditional foods, extruded snacks	Moderate	[91]
Common bean peptides (GLTSK, LSGNK, etc.)	*Phaseolus vulgaris*	Cell cycle regulation; intrinsic apoptosis [80]	Canned beans, soups, stews	Moderate	[80]
Soybean meal peptides (PRPIPFPRPQP, etc.)	Soybean (*Glycine max*)	Cytotoxicity against glioblastoma; selectivity [81]	Protein-rich by-product flours, supplements	Moderate	[81]
Hemp bioactive peptides	Hemp seeds	ROS induction; Akt/GSK3β/β-catenin inhibition [83]	Seed-based milks, protein powders, snacks	Moderate	[83]
Walnut peptides (PISLKSE, VSLP, SHTLP)	Walnut (*Juglans regia*)	CASP3 activation; MMP9 inhibition [97]	Nut-based products, bakery, confectionery	High (stable peptides)	[97]
Perilla seed peptide PSO3 (SGP VGLW)	Perilla seed	Cytotoxicity against glioma, lung, colon, liver [98]	Oilseed meals, Asian cuisine, supplements	Not specified	[98]

## Data Availability

This is a comprehensive review manuscript and all the data is contained within this article.
